# In Silico and In Vitro Insights into the Pharmacological Potential of *Pouzolzia zeylanica*

**DOI:** 10.3390/molecules31020357

**Published:** 2026-01-20

**Authors:** Nguyen Anh Hung, Vu Thi Thu Le, Nguyen Viet Hung, Ha Thi Minh Tam, Nguyen Ngoc Linh, Nguyen Quang Hop, Nguyen Thi Hanh, Do Tien Lam

**Affiliations:** 1 Faculty of Chemistry, Hanoi Pedagogical University 2, Phuc Yen 15000, Vietnam; 2 Faculty of Basic Sciences, Thai Nguyen University of Agriculture and Forestry (TNU), Quyet Thang, Thai Nguyen 24119, Vietnam; 3 Ethnic Institute, Tay Mo, Hanoi 10072, Vietnam; 4 Preschool Education, Hanoi Pedagogical University 2, Phuc Yen 15000, Vietnam; 5 Faculty of Training Bachelor of Practice, Thanh Do University, Kim Chung, Hanoi 10072, Vietnam; 6 Center for High Technology Research and Development, Vietnam Academy of Science and Technology, 18 Hoang Quoc Viet, Nghia Do, Hanoi 10072, Vietnam; 7 Institute of Chemistry, Vietnam Academy of Science and Technology, 18 Hoang Quoc Viet, Nghia Do, Hanoi 10072, Vietnam; 8 Faculty of Chemistry, Graduate University of Science and Technology, Vietnam Academy of Science and Technology, 18 Hoang Quoc Viet, Nghia Do, Hanoi 10072, Vietnam

**Keywords:** *Pouzolzia zeylanica*, in silico, anti-inflammatory, antioxidant, molecular docking

## Abstract

The present study involves the isolation, structural elucidation, and biological evaluation of eight compounds from *Pouzolzia zeylanica*. From the *n*-hexane and ethyl acetate extracts of the plant, eight compounds were successfully isolated and identified: oleanolic acid (**1**), ursolic acid (**2**), 2*α*-hydroxyursolic acid (**3**), 3*β*-O-acetyl-12-oleanen-28-oic acid (**4**), 5-hydroxy-6,7-dimethoxyflavanone (**5**), 4′-methoxytectochrysin (**6**), 3,4′,5,7-tetrahydroxyflavanone-3-O-L-rhamnopyranoside (**7**), and 3,3′,5,5′,7-pentahydroxyflavanone-3-O-L-rhamnopyranoside (**8**). These compounds were evaluated for in vitro antioxidant activity using the 1,1-diphenyl-2-picrylhydrazyl (DPPH) and lipid peroxidation inhibition (TBARS) assays, as well as anti-inflammatory activity via inhibition of nitric oxide (NO) production and the secretion of pro-inflammatory cytokines tumour necrosis factor-alpha (TNF-*α*) and interleukin-6 (IL-6) in RAW 264.7 macrophages. It was observed that compound **3** exhibited the strongest antioxidant activity with IC_50_ values of 18.52 ± 1.50 µM (DPPH) and 10.34 ± 0.93 µM (TBARS), whereas compounds **2**, **5**, and **6** showed moderate to weak effects. Meanwhile, compound **8** demonstrated the most potent anti-inflammatory effect with IC_50_ values of 16.25 ± 0.95 µM (NO inhibition), 12.97 ± 0.88 µM (TNF-*α* inhibition), and 22.52 ± 1.98 µM (IL-6 inhibition). Furthermore, in silico approaches were employed, including density functional theory (DFT) calculations to predict the antioxidant mechanisms of compounds **1** and **3** and molecular docking to assess the cyclooxygenase-2 (COX-2) and phosphodiesterase-4B (PDE4B) inhibitory potentials of compounds **4**, **7**, and **8**. Computational results aligned well with experimental data, supporting the potential of these compounds as natural antioxidant and anti-inflammatory agents.

## 1. Introduction

*P. zeylanica* (L.) Benn. (Urticaceae) is a traditional medicinal herb widely recognized for its therapeutic potential, particularly its antioxidant and anti-inflammatory properties [1,2,3,4,5]. Previous phytochemical analyses have demonstrated that extracts of *P. zeylanica* contain substantial amounts of phenolic compounds, flavonoids, and anthocyanins, which correlate with significant free radical scavenging activity and ferric reducing power [6,7,8,9,10]. In parallel, anti-inflammatory evaluations have shown that plant extracts effectively inhibit key inflammatory mediators and pathways, including NO production and pro-inflammatory cytokine secretion in cellular models, while also reducing edema in animal studies [10,11,12,13,14,15]. Despite these promising findings at the extract level, a critical research gap persists: the specific chemical constituents responsible for these bioactivities remain largely unidentified and their molecular mechanisms of action are poorly understood. This represents a significant limitation in advancing *P. zeylanica* from traditional use toward evidence-based therapeutic development [16,17,18,19,20].

Modern natural product research increasingly incorporates computational approaches to accelerate bioactive compound discovery and mechanistic elucidation [21,22,23]. For antioxidant investigation, DFT calculations provide quantum-chemical insights by evaluating thermodynamic parameters that predict radical scavenging potential through different mechanistic pathways [24,25,26,27]. For anti-inflammatory activity assessment, molecular docking and dynamics (MD) simulations enable virtual screening against validated protein targets involved in inflammatory cascades, such as COX-2 and PDE4B, offering predictions about binding interactions and complex stability [28,29,30,31]. The integration of these in silico methods with classical phytochemical isolation and in vitro bioassays creates a powerful, multi-dimensional strategy to not only identify lead compounds but also to generate testable hypotheses regarding their molecular mechanisms [32,33,34,35,36].

To address the identified research gaps and leverage contemporary methodological integration, this study was designed with the following specific objectives: (i) to isolate and structurally characterize the major chemical constituents from *P. zeylanica*; (ii) to evaluate their in vitro antioxidant activity using DPPH and TBARS assays; (iii) to assess their anti-inflammatory potential by measuring inhibition of NO, TNF-*α*, and IL-6 in LPS-stimulated RAW 264.7 macrophages; (iv) to employ DFT calculations to elucidate the antioxidant mechanisms of the most active compounds; (v) to perform molecular docking and MD simulations against COX-2 and PDE4B to explore their potential anti-inflammatory targets and binding modes. Through this comprehensive approach, our work aims to establish a detailed phytochemical and pharmacological profile of *P. zeylanica*, providing a robust scientific foundation for its traditional uses and highlighting specific compounds worthy of further development as natural antioxidant and anti-inflammatory agents.

## 2. Results and Discussions

### 2.1. Chemical Constituents of P. zeylanica

**Figure 1:**
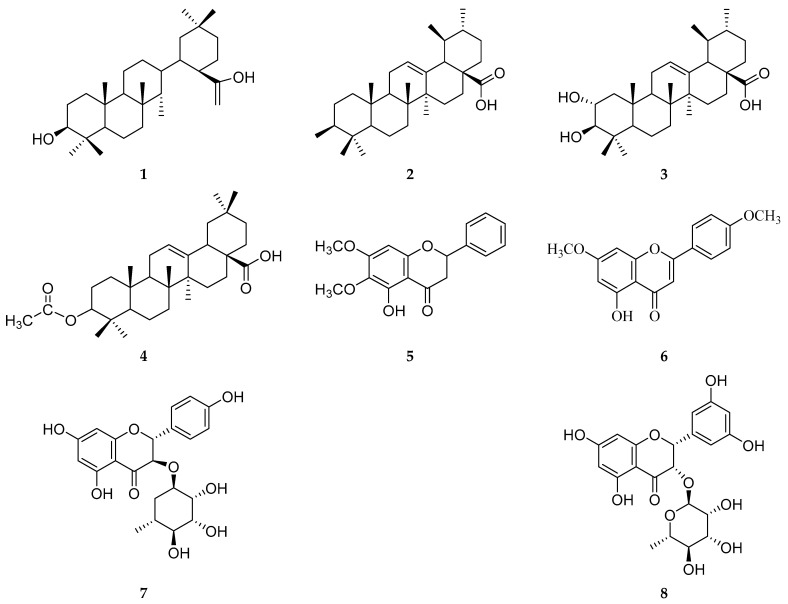
Chemical structure of compounds **1**–**8.**

Phytochemical investigation of *P. zeylanica* led to the isolation of eight known compounds (**1**–**8**). Their structures were determined by extensive NMR analysis (^1^H, ^13^C) and comparison with published data (Figure 1). Compounds **1**–**4** are pentacyclic triterpenoids, while **5**–**8** are flavonoids [37,38,39,40,41,42,43,44].

Compound **1** was identified as oleanolic acid based on characteristic NMR signals for seven methyl singlets (δ_H_ 0.75–1.13), a hydroxylated methine at C-3 (δ_H_ 3.23, δ_C_ 79.1), a Δ^12^ double bond (δ_H_ 5.27, H-12; δ_C_ 122.6 and 143.6), and a carboxylic acid at C-28 (δ_C_ 183.2) [37]. Compound **2** was determined to be ursolic acid, showing seven methyls (including two doublets), a CH-OH at δ_H_ 3.17 (C-3, δ_C_ 79.0), an olefinic proton at δ_H_ 5.24 (H-12), and a carbonyl at δ_C_ 180.6, consistent with an ursane skeleton [37]. Compound **3** exhibited a structure similar to **2**, with additional hydroxylation at C-2, evidenced by signals at δ_H_ 3.40 (H-2)/δ_C_ 67.1 and δ_H_ 2.73 (H-3)/δ_C_ 82.2, along with a carboxyl at δ_C_ 178.2, identifying it as 2*α*-hydroxyursolic acid [38,39]. Compound **4** was characterized as 3*β*-O-acetyl-12-oleanen-28-oic acid. Its NMR data closely matched **1**, except for the acetyl substitution at C-3, indicated by a downfield shifted H-3 (δ_H_ 4.50, δ_C_ 81.0) and an additional carbonyl at δ_C_ 170.9 [40].

Among the flavonoids, compound **5** was identified as 5-hydroxy-6,7-dimethoxyflavanone, with key signals including two methoxyls (δ_H_ 3.87, 3.88), a methylene at C-3 (δC 45.7), and a carbonyl at δ_C_ 189.0 (C-4) [41]. Compound **6** was established as 4′-methoxytectochrysin, displaying meta-aromatic protons (δ_H_ 6.49, H-8; 6.37, H-6), an intramolecular H-bonded hydroxyl (δ_H_ 12.09), two methoxyls, and a carbonyl at δ_C_ 182.5 [41,42]. Compound **7** was determined to be 3,4′,5,7-tetrahydroxyflavanone-3-O-L-rhamnopyranoside. Key features included B-ring protons (δ_H_ 7.38, H-2′/6′; 6.87, H-3′/5′), flavanone protons H-2/H-3 (δ_H_ 5.16, 4.63), and a sugar anomeric proton at δ_H_ 4.04 (δ_C_ 102.2) [43]. Finally, compound **8** was identified as 3,3′,5,5′,7-pentahydroxyflavanone-3-O-L-rhamnopyranoside, supported by signals for five aromatic singlets, hydroxyl protons at δ_H_ 11.75 and 8.85, flavanone protons H-2/H-3 (δ_H_ 5.54, 4.21), and a rhamnosyl anomeric proton at δ_H_ 4.76 (δ_C_ 98.8) [44].

The isolation of common triterpenoids like oleanolic acid (**1**) and ursolic acid (**2**) from *P. zeylanica* aligns with previous reports on the Urticaceae family, which is known as a rich source of such bioactive compounds [37]. However, the identification of **3** (2*α*-hydroxyursolic acid) and **4** (acetylated derivative) in this species is less common and adds to the phytochemical profile of *P. zeylanica*. The presence of flavonoids, particularly the glycosylated derivatives **7** and **8**, is significant. Flavonoid glycosides are often associated with enhanced solubility and biological activities in medicinal plants. The co-occurrence of both aglycone (**5**, **6**) and glycosylated flavonoids (**7**, **8**) suggests a diverse biosynthetic pathway in this plant. This chemical profile provides a material basis for explaining the traditional uses and the observed pharmacological activities of *P. zeylanica*.

### 2.2. Antioxidant Activity

#### 2.2.1. In Vitro Antioxidant Evaluation

**Figure 2:**
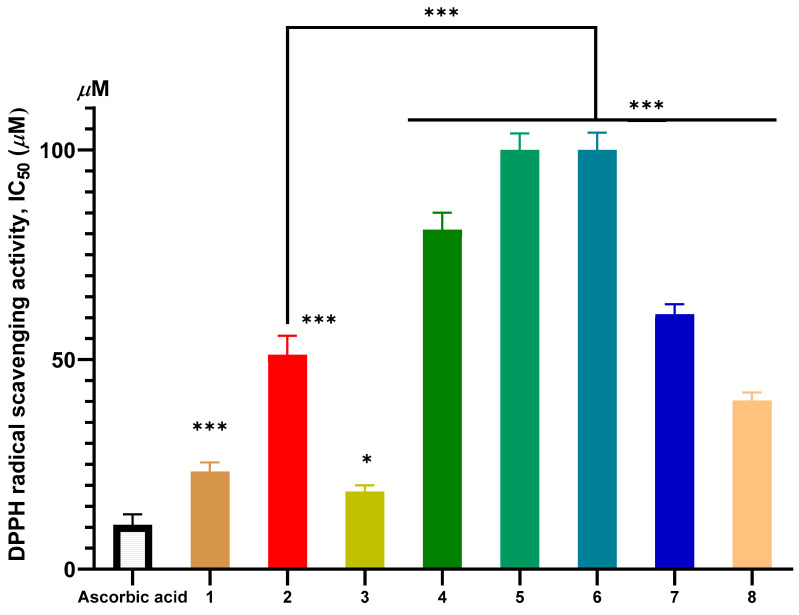
Antioxidant activity of compounds **1**–**8** determined by the DPPH assay. Data are presented as mean ± SD (*n* = 3). Statistical significance was determined by one-way ANOVA followed by Tukey’s post hoc test. Asterisks denote significant differences versus the control group: * *p* < 0.05, *** *p* < 0.001.

**Figure 3:**
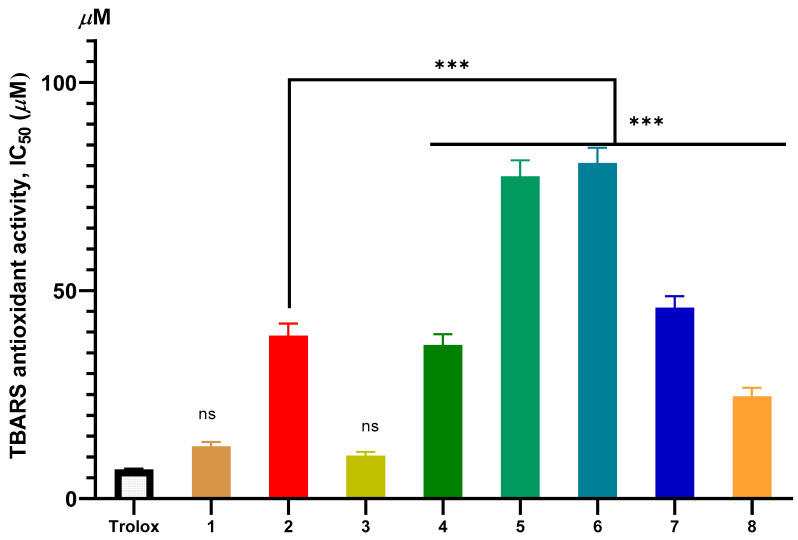
Results of testing the in vitro antioxidant activity by TBARS method of compounds **1**–**8**. Data are presented as mean ± SD (*n* = 3). Statistical significance was determined by one-way ANOVA followed by Tukey’s post hoc test. Asterisks denote significant differences versus the control group: *p* < 0.001, ns: not statistically significant.

The dose–response curves depicting the antioxidant activities of the isolated compounds (**1**–**8**) are shown in Figure 2, Figure 3 and Figure S2. The antioxidant potential of the isolated compounds was assessed using two complementary assays: DPPH radical scavenging (measuring hydrogen-donating ability) and TBARS inhibition (evaluating protection against lipid peroxidation). The standard positive controls, ascorbic acid for the DPPH assay (IC_50_ = 10.55 ± 2.54 µM) and Trolox for the TBARS assay (IC_50_ = 12.53 ± 1.10 µM), demonstrated potent activity as expected, validating the reliability of the assay systems (also included in Figure 2 and Figure 3, respectively).

The triterpenoid compounds (**1**–**4**) exhibited significantly stronger antioxidant activity compared to the flavonoids (**5**–**8**) (Figure 2 and Figure 3). Compound **3** was the most potent, with IC_50_ values of 18.52 ± 1.50 µM (DPPH) and 10.34 ± 0.93 µM (TBARS). This was followed by compound **1** (IC_50_: 23.32 ± 2.16 µM for DPPH; 12.53 ± 1.10 µM for TBARS). In contrast, the simple flavonoids **5** and **6** showed negligible activity, while the polyhydroxylated flavonoid glycosides **7** and **8** displayed only moderate effects.

The superior antioxidant activity of the triterpenoids, particularly compound **3**, can be attributed to their structural features. The presence of both carboxylic acid and hydroxyl groups provides active sites for HAT and SET, which are key mechanisms for neutralizing free radicals. The significantly enhanced activity of **3** over its parent compound, ursolic acid (**2**), underscores the critical role of the additional 2α-hydroxyl group. This observation aligns with established structure-activity relationship (SAR) studies on pentacyclic triterpenoids, which suggest that increased hydroxylation enhances radical scavenging capacity by stabilizing the resulting radical intermediates [45,46].

Conversely, the weak activity of flavonoids **5** and **6** is due to the absence of free phenolic-OH groups, as these positions are substituted by methoxy groups. The fundamental importance of free phenolic hydroxyls for the antioxidant activity of flavonoids is well-documented [47,48,49]. The moderate activity of the glycosylated flavonoids **7** and **8**, despite their high hydroxyl count, may be explained by the presence of the glycosyl moiety. Glycosylation can sterically hinder the interaction of phenolic-OH groups with radicals or reduce membrane permeability in the lipophilic environment of the TBARS assay.

The potent DPPH scavenging activity of compound 3 (IC_50_ = 18.52 µM) is notable and comparable to several common natural antioxidants. For instance, its activity is lower than that of quercetin (IC_50_: 7.3 µM), and within a competitive range compared to α-tocopherol (IC_50_: 12.3 µM) and caffeic acid (IC_50_: 16.2 µM) as reported in similar assay systems [50,51]. This comparative analysis situates compound **3** as a highly effective natural antioxidant. Our findings not only confirm prior reports on the strong DPPH activity of *P. zeylanica* extracts [1,5] but also identify **3** as a key phytochemical contributor to this effect.

#### 2.2.2. The Antioxidant Activity in Silico

To elucidate the molecular basis of the antioxidant activity observed in vitro, in silico analyses were performed on compounds **1** and **3**.

**Figure 4:**
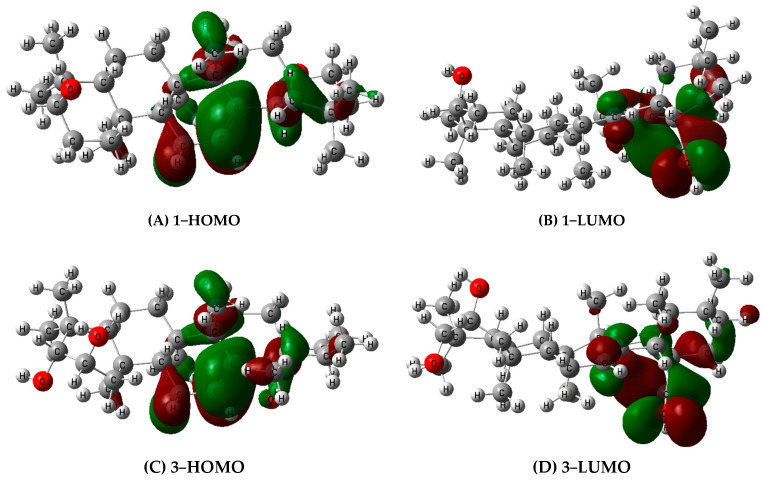

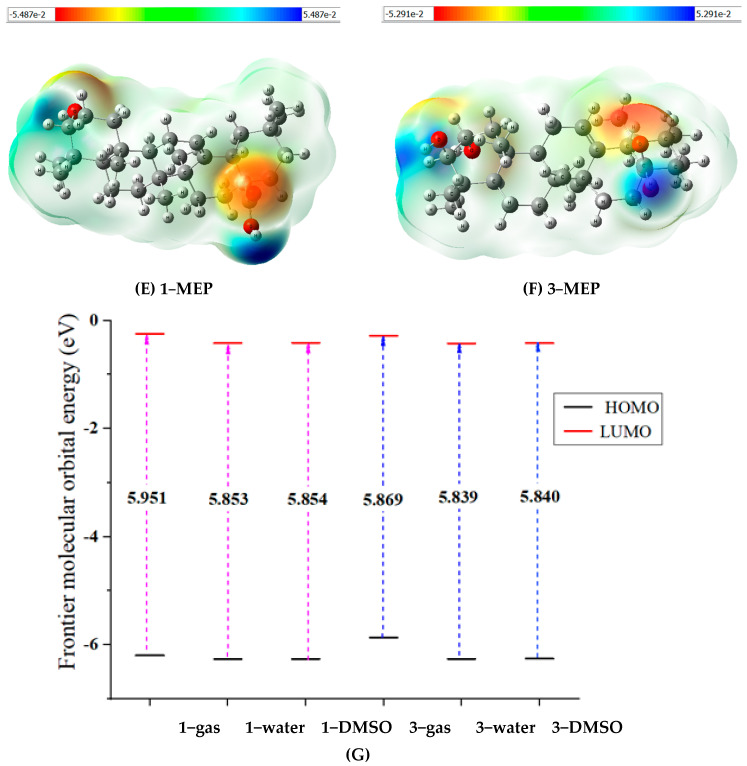
FMO surfaces, MEP maps, and orbital energy levels for compounds **1** and **3**. (**A**,**B**) The HOMO and LUMO isosurfaces (isovalue = 0.02 a.u.) for compound **1**. (**C**,**D**) The HOMO and LUMO isosurfaces for compound **3**. (**E**,**F**) MEP maps plotted on the electron density surface (isovalue = 0.0004 a.u.) for compounds **1** and **3**, respectively. Red regions indicate negative electrostatic potential (electron-rich), blue regions indicate positive potential (electron-deficient). (**G**) Comparison of the HOMO-LUMO energy gap for compounds **1** and **3** in different phases (gas, water, DMSO). All calculations were performed at the B3LYP/6-31+G(d,p) level of theory.

The electronic distributions of compounds **1** and **3** were examined using the FMO analysis. The FMO images in the three examined media for each compounds were comparable (Figure 4). Electron concentration was mostly in a double bond, with a minimal concentration in the **1**-OH group, as shown in the **1**-HOMO neutral picture. Comparably, a high electron density in a double bond is linked to a **3**-HOMO neutral image. The two compounds under study exhibit identical LUMO models, whereby the 1,3-OH group lacks electrons. A smaller band gap energy (E_gap_ = E_L_ − E_H_) indicates higher reactivity. As shown in Figure 4, the Egap values of the two compounds under study in DMSO and water with values from 5.839 to 5.854 eV were consistently lower than those in the gas phase, ranging from 5.869 to 5.951 eV. This implies that the solvent environment facilitates the radical scavenging reaction by lowering the reaction energy. The analysis of FMO and molecular electrostatic potential (MEP) maps is a well-established computational approach for predicting chemical reactivity and identifying sites susceptible to radical attack in antioxidant studies [52,53].

**Figure 5:**
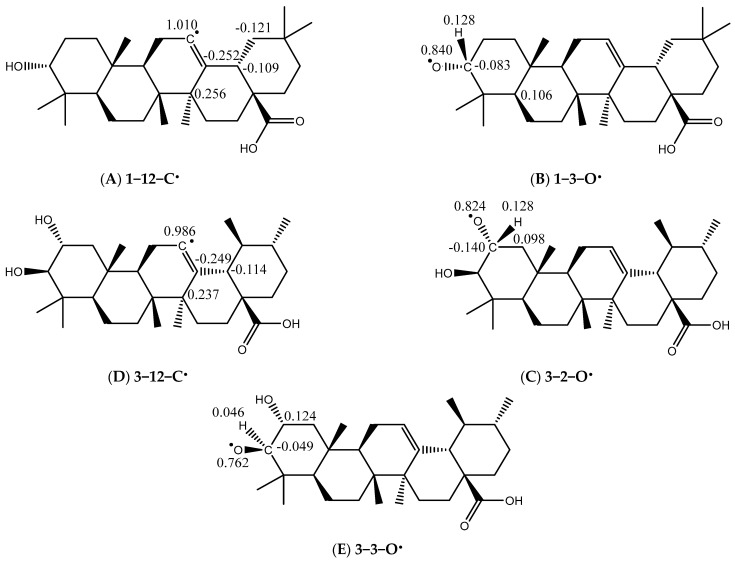
Spin density distribution (isosurface value = 0.002 a.u.) of the key radical intermediates derived from compounds **1** and **3**. Panels show the radicals formed after hydrogen abstraction from specific sites: (**A**) Radical generated from a C–H bond (e.g., at C-12) of compound **1** (**1-C**•). (**B**) Radical generated from the O–H bond at the C-3 position of compound **1** (**1-O**•). (**C**) Radical generated from an O–H bond (e.g., at C-2 or C-3) of compound **3** (**3-O**•). (**D**) Radical generated from a C–H bond of compound **3** (**3-C**•). The more delocalized spin density in the O-centered radicals (**A**,**C**) compared to the C-centered radicals (**B**,**D**) indicates greater stability, supporting the lower BDE values for O–H bonds (see Table 1). Calculations were performed at the B3LYP/6-31+G(d,p) level in the gaseous phase.

The stability of radicals is determined using spin density analysis. The stability of a radical is indicated by the delocalization of its spin density. Greater delocalization leads to a more stable radical intermediate. The radical formed from R-O exhibits greater dispersion compared to the R-C radical (in Figure 5) a phenomenon observed in both compounds **1** and **3**. The degree of spin delocalization is a key indicator of radical stability, where greater dispersion correlates with a more stable radical intermediate and a thermodynamically favorable antioxidant action [54,55].

**Figure 6:**
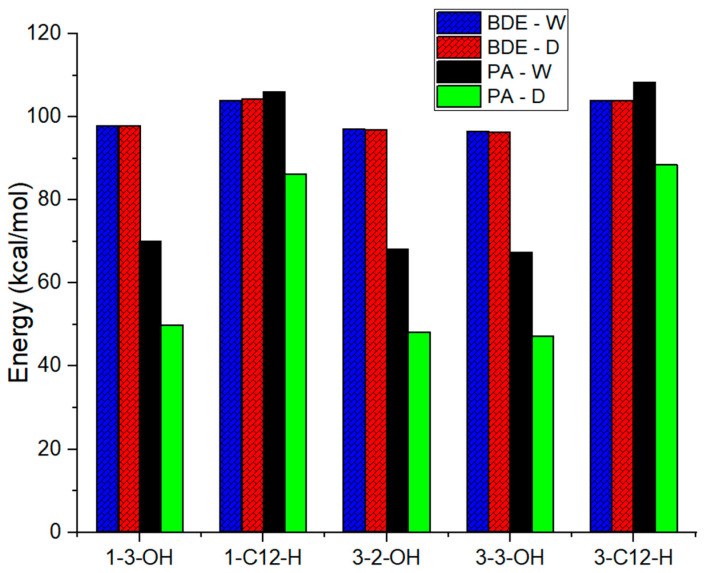
Thermochemical properties including BDE and PA values (in kcal/mol) in water and DMSO at 298.15 K calculated at the B3LYP/6-31+G(d,p) level of theory.

Table 1 and Figure 6 calculate and summarize the intrinsic parameters, which include BDE, PA, and IP, which characterize the FHT, PT, and SET processes, respectively [56]. The simpler capacities to donate hydrogen, protons, and electrons are accordingly described by lower BDE, PA, and IP values. BDE analysis served as the primary foundation for the homolytic H-disruption, which was the formal hydrogen atom transfer (FHAT) mechanism. The enthalpies required to split CH and OH at carbons **1**-C12-H and 1-OH of compound **1** were 104.1–105.0 and 97.8 –98.0 kcal/mol, respectively, at the B3LYP/6-31+G(d,p) level. Meanwhile, the BDE values of 103.9–104.7; 96.5–97.1, and 96.5–96.3 kcal/mol were produced by the rupture of the 3–CH, 3-OH, and 3–OH’ bonds, respectively. The C-H BDE levels were found to be lower than the O-H BDE values in both compounds. In three phases, 3-OH′ has the lowest BDE value (96.3 kcal/mol) in DMSO, whereas **1**-C12-H has the greatest BDE value (105.0 kcal/mol) in gas.

The PT mechanism plays a crucial role in the radical scavenging capability of compounds in different solvents. This activity varies significantly with the environment, being DMSO < water << gas phase. The minimum PA value of 47.4 kcal/mol was determined for the 3-3-OH compound. Additionally, OH groups consistently exhibit lower PA values (from 47.4 to 70.0 kcal/mol) compared to CH groups (from 86.3 to 401.5 kcal/mol) in both compounds analyzed.

The SET mechanisms is characterized by its IP values. The IP enthalpies of the two substances under study exhibit a consistent order: Gas < DMSO < Water. Consequently, the reactivity of solvents to IP decreases, with polar liquids particularly favoring the electron transfer process. Furthermore, it was observed that the IP value of compound **3** is consistently lower than that of compound **1** across all phases.

The favorable antioxidant mechanisms attributed to compounds **1** and **3** are determined by the minimum values of BDE and IP. The FHAT mechanism is favored in the gas phase due to BDE < IP << PA, while the PT mechanism dominates in solvents where PA < BDE < IP. It was also concluded that deprotonation is the most critical step in the PL mechanism, with solvents such as DMSO and water facilitating the ionization process. By comparing the obtained values that are shown in Figure 5, it is notable that the PT process is preferred in DMSO.

The integrated in vitro and in silico approach, combining experimental assays with DFT-based thermodynamic analysis (BDE, PA, IP), provides a powerful framework for elucidating antioxidant mechanisms at the molecular level, as demonstrated in previous studies on natural products [55,56].The computational results provide a robust mechanistic explanation for the experimental data. The low BDE and PA values for the hydroxyl groups, especially in **3**, confirm that HAT and PT are the dominant, thermodynamically favorable pathways for its radical scavenging activity in solution. The even lower PA values in DMSO compared to water suggest that the antioxidant action might be particularly efficient in less polar microenvironments, such as lipid bilayers-a finding consistent with its strong performance in the TBARS assay. The enhanced activity of compounds **3** relative to compounds 1 was computationally validated by its lower BDE, PA, and IP values, emphasizing the role of the 2α-hydroxyl group. This integrated in vitro and in silico approach moves beyond simple activity reporting to offer a predictive model of antioxidant behavior at the quantum chemical level.

### 2.3. Anti-Inflammatory Activity

#### 2.3.1. In Vitro Anti-Inflammatory Evaluation

**Figure 7:**
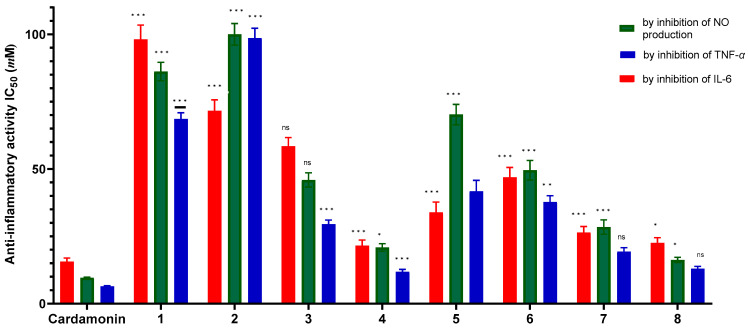
In vitro anti-inflammatory activity of compounds **1**–**8**. Activity was assessed by measuring the inhibition of NO production, TNF-α, and IL-6 in LPS-stimulated RAW 264.7 macrophages. For NO assay, cells were pre-treated with various concentrations of the compounds for 1 h, followed by stimulation with LPS (1 µg/mL) for 24 h. NO levels were measured using the Griess assay. For TNF-α and IL-6 assays, cells were co-treated with the compounds and LPS (1 µg/mL) for 24 h, and cytokine levels were quantified by ELISA. Data are presented as mean ± SD (*n* = 3). Statistical significance was determined by one-way ANOVA followed by Tukey’s post hoc test. Asterisks denote significant differences versus the control group: * *p* < 0.05, ** *p* < 0.01, *** *p* < 0.001; ns: not statistically significant.

The anti-inflammatory potential was evaluated by measuring the inhibition of NO production and the secretion of pro-inflammatory cytokines (TNF-*α*, IL-6) in LPS-stimulated RAW 264.7 macrophages. The positive control consistently showed the strongest activity. Among the tested compounds, Compound **4** (IC_50_ values: 20.86 ± 1.42 µM for NO, 11.9 ± 0.84 µM for TNF-*α*, 21.55 ± 2.06 µM for IL-6) and compound **8** (IC_50_values: 16.25 ± 0.95 µM for NO, 12.97 ± 0.88 µM for TNF-*α*, 22.52 ± 1.98 µM for IL-6) stand out with potent and well-balanced activity across all three parameters. Specifically, compound **4** demonstrated the strongest inhibition of TNF-*α*, while compound **8** showed the most potent inhibition of NO, indicating their potential for broad-spectrum anti-inflammatory effects (Figure 7 and Figure S2).

In contrast, the common free triterpenoic acids compound **1** and compound **2** displayed very weak activity in this model despite their known antioxidant activity. This suggests that their anti-inflammatory mechanism is independent of direct radical scavenging. Notably, the acetylation in Compound **4** completely transformed its activity profile from weak to potent, indicating that its primary mechanism of action likely involves the specific inhibition of pro-inflammatory signaling pathways, rather than direct free radical neutralization.

Compounds **4** and **8** are the two most promising candidates for further investigation. Priority should be given to conducting in silico screening for these two compounds to predict their molecular targets and elucidate their mechanisms of action in inhibiting specific inflammatory signaling pathways, thereby providing a foundation for subsequent in vivo testing.

The dissociation between strong antioxidant and anti-inflammatory activities is a significant finding. While oxidative stress and inflammation are linked, this result suggests that the primary anti-inflammatory mechanism of the active compounds (**4** and **8**) is not merely due to general radical scavenging. The potent activity of compound **4**, an acetylated derivative of oleanolic acid, is particularly intriguing. Acetylation of the 3*β*-OH group dramatically enhanced anti-inflammatory potency compared to its parent compound **1**. This modification likely increases lipophilicity and membrane permeability, allowing better cellular uptake and interaction with intracellular inflammatory targets. The strong, multi-target inhibition by flavonoid glycoside **8** indicates its potential to interfere with upstream signaling pathways (like NF-κB or MAPK) that govern the expression of both iNOS (responsible for NO) and cytokines like TNF-*α* and IL-6. Our in vitro-like cell-based results corroborate and extend previous in vivo findings on *P. zeylanica* extracts reducing paw edema [8,9,10], by pinpointing specific compounds (**4** and **8**) and inflammatory mediators (NO, TNF-*α*, IL-6) involved.

Notably, the weak anti-inflammatory activity observed for compound **1** and **2** in our LPS-induced RAW 264.7 model contrasts with previous studies reporting their potent inhibition of NO and TNF-*α* [57,58,59,60]. This discrepancy may stem from differences in experimental conditions (e.g., LPS concentration, incubation time), compound purity or stereochemistry, or distinct mechanisms of action that are context-dependent. Future investigations, including direct comparative assays and mechanistic studies, are warranted to clarify the anti-inflammatory roles of these triterpenoids.

#### 2.3.2. In Silico Evaluation of Anti-Inflammatory Targets

To explore the potential enzymatic targets, molecular docking and dynamics simulations were performed against two key inflammatory enzymes: COX-2 and PDE4B. Compounds **4**, **7**, and **8** were selected for docking studies due to their potent and balanced anti-inflammatory activity across multiple targets (NO, TNF-*α*, IL-6) in our in vitro assays, while compound **3**, despite strong antioxidant activity, showed weak anti-inflammatory effects.

##### Molecular Docking

**Figure 8:**
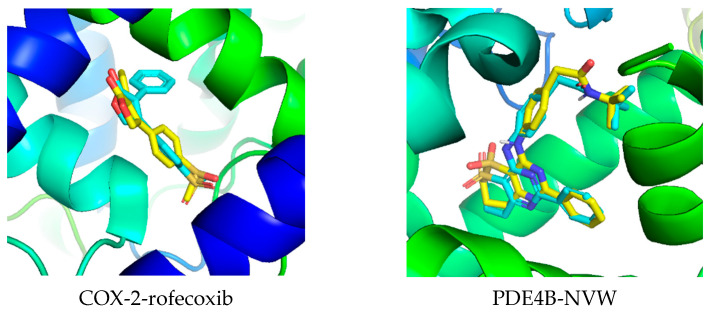
Three-dimensional superimposition of the native ligand (in cyan) and re-docked ligand (in yellow) of the selected complexes. RMSD values: COX-2 = 1.32 Å; PDE4B = 1.58 Å.

First, the molecular docking validation method was performed to evaluate the reliability of the docking protocol. The RMSD value was calculated based on the coordinates of the initial co-crystallized ligand and the re-docked ligand to compare the experimentally determined and theoretically predicted positions. The results showed that for COX-2 and PDE4B was 1.32 Å and 1.58 Å, respectively (Figure 8), both less than 2 Å, indicating that this protocol is suitable for further studies.

In this study, to better understand the potential anti-inflammatory capabilities of compounds **4**, **7**, and **8** against known targets (the enzymes COX-2 and PDE4B), molecular docking studies were performed. The binding affinities of compounds **4**, **7**, and **8** to the COX-2 protein ranged from −6.265 to −7.17 kcal/mol, while for the PDE4B protein, these values ranged from −6.146 to −9.74 kcal/mol, as shown in Table 2. The reference compounds, rofecoxib and NVW, had binding affinities of −8.9 and −12.05 kcal/mol, respectively. These results indicate that compounds **4**, **7**, and **8** have a better binding affinity with PDE4B. The binding modes of the ligands and proteins are illustrated in Figure 5 and Figure 6. 

**Figure 9:**
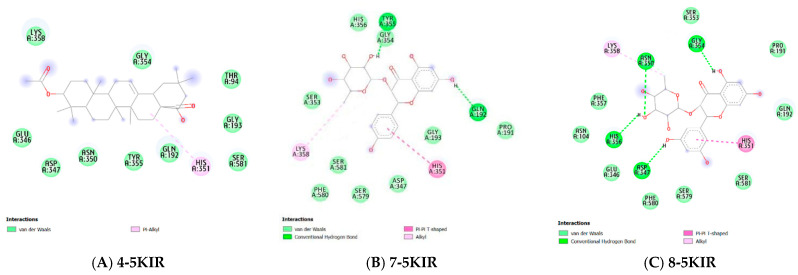
Molecular interactions of the studied compounds with the COX-2 active site (PDB: 5KIR). (**A**) Three-dimensional binding pose and two-dimensional interaction diagram of compound **4**. (**B**) Binding pose and interaction diagram of compound **7**. (**C**) Binding pose and interaction diagram of compound **8**. Hydrogen bonds are shown as green dashed lines, while hydrophobic interactions (π-alkyl, π-π T-shaped) are represented by pink/light blue dashed lines. Key interacting residues are labeled.

Compound **4** forms a pi-alkyl interaction with the amino acid residue His351 of the COX-2 enzyme. Compound **7** forms hydrogen bonds with the COX-2 enzyme at the amino acid residues Tyr355, Asn350, and Gln192. Additionally, hydrophobic interactions are found between this ligand and His351 and Lys358 through pi-pi T-shaped and alkyl interactions, respectively. These hydrophobic interactions are also observed in the complex of compound **8** with the COX-2 protein. Compound **8** establishes hydrogen bonds with the residues Tyr355, Gly354, Asn350, His356, and Asp347 (Figure 9).

**Figure 10:**
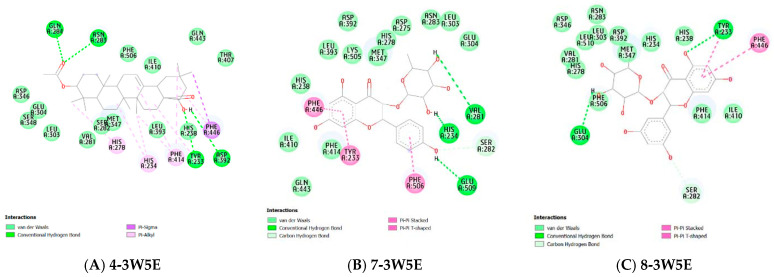
Molecular interactions of the studied compounds with the PDE4B active site (PDB: 3W5E). (**A**) Three-dimensional binding pose and two-dimensional interaction diagram of compound **4**. (**B**) Binding pose and interaction diagram of compound **7**. (**C**) Binding pose and interaction diagram of compound **8**. Hydrogen bonds and key hydrophobic interactions (π-π, π-sigma) are indicated. The reference inhibitor (NVW) binding mode is shown for comparison in relevant panels.

For the enzyme PDE4B, the interactions of compounds **4**, **7**, and **8** are observed in Figure 9. Specifically, compound 4 forms hydrogen bonds with the amino acid residues Gln284, Asn283, Tyr233, and Asp392. Additionally, this compound exhibits hydrophobic interactions with the amino acid residues His278, His234, and Phe414 through pi-alkyl interactions and with Phe446 through a pi-sigma interaction. Compound **7** forms hydrogen bonds with the residues His234, Val281, and Glu509, and engages in pi-pi interactions with the residues Phe446, Tyr233, and Phe506. Meanwhile, compound 8 establishes pi-pi interactions with the residues Tyr233 and Phe446, and also forms two hydrogen bonds with Glu304 and Tyr233 (Figure 10, Table 2).

Molecular docking was employed in this study primarily to propose plausible molecular targets (COX-2, PDE4B) and visualize potential binding modes of the active compounds, rather than to provide a quantitative prediction of inhibitory potency. The docking scores offer a relative ranking and qualitative insight into binding affinity, and small differences in scores should not be overinterpreted. The experimental IC_50_ values from cell-based assays remain the definitive measure of anti-inflammatory activity

##### Molecular Dynamics

**Figure 11:**
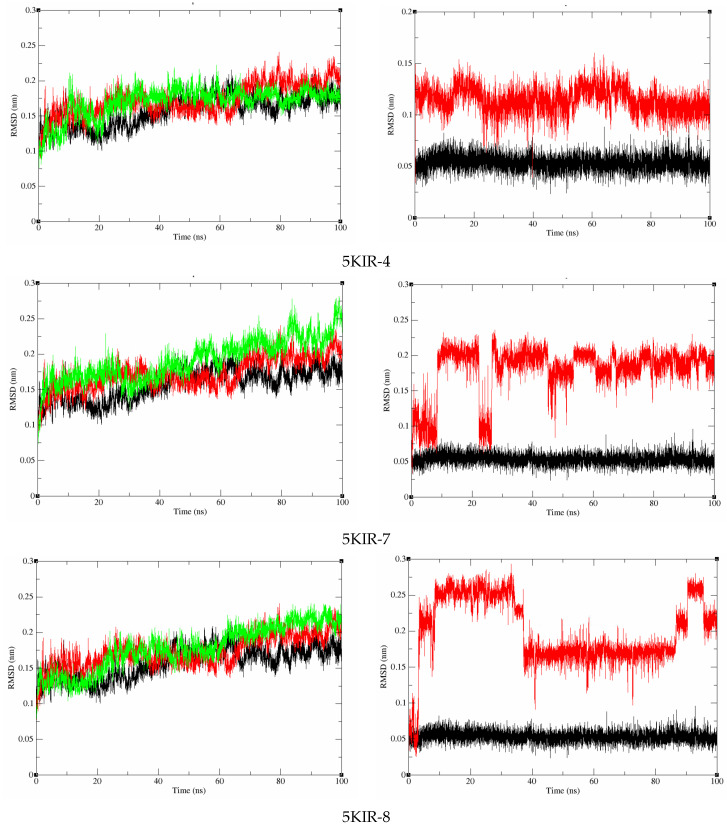
RMSD of the protein backbone and ligand in complex with the COX-2 protein during the 100 ns MD course.

**Figure 12:**
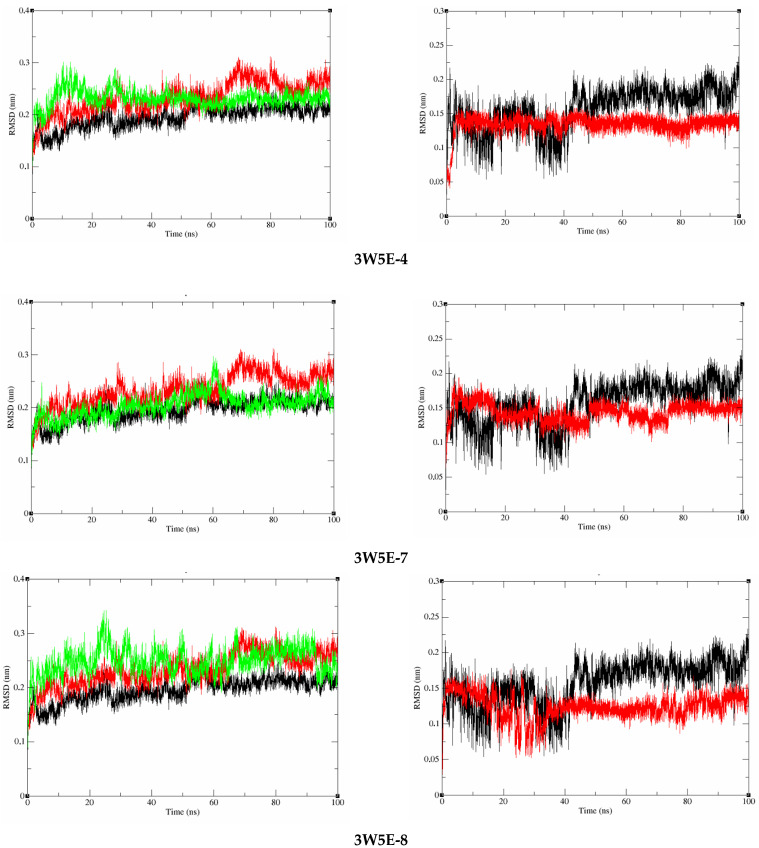
RMSD of the protein backbone and ligand in complex with the PDE4B protein during the 100 ns MD course.

The 100 ns MD simulations were conducted to assess the stability of the docked complexes between the target proteins (COX-2, PDE4B) and the most promising compounds (**4**, **7** and **8**). The stability observed in the RMSD trajectories (Figure 11 and Figure 12) confirms that the binding poses predicted by docking are physically plausible and persistent in a dynamic environment. It should be noted that the scope of this MD analysis was to confirm overall complex stability. Future studies employing extended simulations, combined with detailed analyses of interaction fingerprints (e.g., hydrogen bond occupancy, binding site RMSF) and end-point free energy calculations (e.g., MM-GBSA), will provide a more quantitative and energetically detailed understanding of the binding mechanisms.

To examine whether there are any conformational changes in the COX-2 and PDE4B proteins after complex formation with the studied compounds and to compare them with the reference compound, RMSD analysis was conducted on the 100 ns MD simulations (Figure 11 and Figure 12). As observed in Figure 12, the average RMSD values for the COX-2 complexes with compounds **4**, **7**, and **8** are 0.169959 nm, 0.19137 nm, and 0.17799 nm, respectively. Meanwhile, the RMSD values for the PDE4B complexes with these compounds are 0.231477127 nm, 0.204981093 nm, and 0.248468489 nm (Figure 11). The RMSD analysis indicates that the proteins in the examined complexes achieved stable conformations during the simulations, as no significant changes were observed.

For the ligands in the complexes, the average RMSD values of compounds **4**, **7**, and **8** complexed with COX-2 are 0.114364 nm, 0.179394 nm, and 0.199679 nm, respectively. As shown in Figure 12, compound **4** exhibits the most stable structure when interacting with COX-2 compared to the other compounds. On the other hand, compound **8** shows significant changes between 4-40 ns and 90 ns to the end of the simulation, indicating lower stability when binding to COX-2. When complexed with PDE4B, the average RMSD values range from 0.123987669 nm to 0.134742 nm. Notably, compounds **4**, **7**, and **8** have lower RMSD values compared to the reference compound NWM, indicating greater stability. These findings confirm that compounds **4**, **7**, and **8** bind tightly and do not affect the structural stability of the PDE4B protein (Figure 12).

The in silico results provide a plausible mechanistic hypothesis to explain the potent in vitro anti-inflammatory activity observed for compounds **4** and **8**. Notably, both compounds demonstrated strong and stable binding to PDE4B in our MD simulations. As PDE4 is a key enzyme responsible for degrading cAMP, its inhibition leads to elevated intracellular cAMP levels and the subsequent downregulation of multiple pro-inflammatory pathways. This proposed mechanism aligns well with the experimental data, where compounds **4** and **8** effectively suppressed the production of multiple inflammatory mediators (NO, TNF-*α*, and IL-6) in LPS-stimulated RAW 264.7 macrophages. The specific interactions observed with critical PDE4B active site residues, such as Tyr233 and Phe446, are consistent with the binding patterns of known inhibitors, further supporting this hypothesis. While also showing binding to COX-2, albeit with weaker affinity, the primary anti-inflammatory action of these compounds appears to be preferentially mediated through PDE4B inhibition. This target specificity offers a rational explanation for why potent antioxidants like compounds **1** and **3** exhibited only weak activity in our anti-inflammatory assays. The integration of docking (for binding pose prediction) and MD simulations (for complex stability assessment) strengthens the credibility of these computational predictions. However, it is crucial to emphasize that these remain in silico hypotheses. The proposed inhibition of COX-2 and PDE4B requires direct experimental validation through future in vitro enzymatic assays to conclusively confirm these mechanisms of action.

## 3. Materials, Methods and Experimental

### 3.1. Plant Materials

A botanical specimen of *P. zeylanica* (L.) Benn. from the family Urticaceae was collected in Tam Dao town, Vinh Phuc province, Vietnam (GPS coordinates: 21°26′21″ N 105°35′58″ E), in October 2022. The specimen was identified by Dr. Nguyen Quoc Binh of the Vietnam Museum of Nature, Vietnam Academy of Science and Technology. The voucher specimen, designated as PZ22.10.12.VP, is deposited at the Vietnam Museum of Nature.

### 3.2. Methods of Isolation and Structure Determination of Compounds

The isolation and purification procedures followed conventional chromatographic techniques commonly used in natural product chemistry. For thin layer chromatography (TLC), pre-coated silica gel 60 F_254_ plates (0.25 mm, Merck, Darmstadt, Germany) were used. Column chromatography (CC) was performed on silica gel (Kieselgel 60, 70–230 mesh and 230–400 mesh, Merck, Darmstadt, Germany) and RP-18 gel (30–50 μm, Fuji Silysia Chemical Ltd., Aichi, Japan). Size-exclusion chromatography was carried out using Sephadex LH-20 (Merck, Darmstadt, Germany). Nuclear magnetic resonance (NMR) spectra were recorded on a Bruker Avance III 500 MHz spectrometer (Bruker Biospin, Faellanden, Switzerland). The structures of the isolated compounds were determined through the analysis of one-dimensional (^1^H-, ^13^C-NMR, DEPT) and two-dimensional (HMBC, HSQC) NMR spectra, with comparisons to literature data.

### 3.3. Experiment and Separation

*P. zeylanica* sample has been studied using the following standard procedure: The aerial parts (leaves and stems) were wilted, cut into small pieces, dried, and then ground to obtain 3000 g of coarse powder. After that, the sample was extracted three times with methanol using an ultrasonic machine. The combined extracts were concentrated under reduced pressure at a temperature below 323 K to afford the total methanol extract (**PZ**, 220 g). Water was added to the total methanol extract, and the extraction was distributed as *n*-hexane and ethyl acetate, respectively. The extraction includes 63 g of *n*-hexane (**PZH**), 55 g of ethyl acetate (**PZE**), and 101 g of water (**PZW**).

The *n*-hexane extract (**PZH**, 60 g) was roughly separated on a silica gel CC using elution of *n*-hexane-ethyl acetate (HE 99:1 → 1:1) to yield five fractions (**PZH1**–**PZH5**). The **PZH2** fraction (6.2 g) was separated on a silica gel CC eluted with *n*-hexane-ethyl acetate (99:1 → 30:1) to yield four fractions (**PZH2.1**–**PZH2.4**). The **PZH2.3** fraction was recrystallized in acetone to yield compound **1** (oleanolic acid; 9.8 mg). The **PZH3** fraction (3.5 g) was separated on a silica gel CC eluted with *n*-hexane-ethyl acetate (50:1 → 5:1) to obtain three small fractions (**PZH3.1**–**PZH3.3**). The **PZH3.2** fraction was recrystallized in acetone to yield compound **2** (ursolic acid; 8.9 mg). The **PZH4** fraction (2.9 g) was separated by RP18 CC using elution of methanol-water (3:7 → 7:3) to produce four fractions (**PZH4.1**–**PZH4.4**). The **PZH4.3** fraction was recrystallized with acetone solvent to give compound **3** (2*α*-hydroxyursolic acid; 5.5 mg). The **PZH4.4** fraction was recrystallized with acetone solvent to give compound **4** (3*β*-O-acetyl-12-oleanen-28-oic acid; 7.2 mg).

The ethyl acetate extract (**PZE**, 50 g) was roughly separated by silica gel CC using elution of dichloromethane-methanol (99:1 → 1:1) to yield five fractions (**PZE1**–**PZE5**). The **PZE1** fraction (4.5 g) was separated by RP18 CC using elution of methanol-water (3:7 → 7:3) to produce three fractions (**PZE1.1**–**PZE1.3**). The **PZE1.2** fraction was separated further using Sephadex LH-20 CC and eluting with methanol to give compound **5** (5-hydroxy-6,7-dimethoxyflavanone; 6.5 mg). The **PZE1.1** fraction was recrystallized with acetone solvent to give compound **6** (4′-methoxytectochrysin; 12.8 mg). The **PZE2** fraction (7.0 g) was separated by silica gel CC using elution of dichloromethane-methanol (50:1 → 10:1) to yield three fractions (**PZE2.1**–**PZE2.3**). The **PZE2.2** fraction was separated further using Sephadex LH-20 CC and eluting with methanol to give compound **7** (3,4′,5,7-tetrahydroxyflavanone-3-O-L-rhamnopyranoside; 8.2 mg). The **PZE3** fraction (3.3 g) was separated by RP18 CC using elution of methanol-water (3:7 → 7:3) to produce two fractions (**PZE3.1**–**PZE3.2**). The **PZE3.2** fraction was recrystallized with acetone solvent to give compound **8** (3,3′,5,5′,7-pentahydroxyflavanone-3-O-L-rhamnopyranoside; 10.4 mg). Detailed spectral data (NMR) for compounds **1**–**8** are available in the Supplementary Materials (Figure S1).

### 3.4. Computational Methods

#### 3.4.1. DFT Calculation

The Gaussian 09 package was employed for all quantum chemical calculations [22]. Geometries of neutral, anionic, and radical species of the studied compounds were fully optimized using the B3LYP functional with the 6-31+G(d,p) basis set. Solvent effects (water and DMSO) were incorporated using the IEF-PCM solvation model.

Thermodynamic Parameters for Antioxidant Mechanism Analysis:

Three key thermodynamic parameters were calculated to evaluate the feasibility of different antioxidant mechanisms:

Bond dissociation enthalpy (BDE): Calculated using Equation (1), BDE represents the energy required for homolytic cleavage of the O–H bond. Lower BDE values indicate greater ease of hydrogen atom transfer (HAT), a primary radical scavenging pathway.

BDE = H(R^•^) + H(H^•^) − H(RH) (1)

Ionization potential (IP): Calculated using Equation (2), IP represents the energy required to remove one electron from the molecule. Lower IP values facilitate single electron transfer (SET) mechanisms.

IP = H(RH^+•^) + H(e^−^) − H(RH) (2)

Proton affinity (PA): Calculated using Equation (3), PA represents the enthalpy change for deprotonation. Lower PA values favor proton transfer (PT) mechanisms, often involved in sequential proton-loss electron transfer (SPLET).

PA = H(R^−^) + H(H^+^) − H(RH) (3)

Additionally, frontier molecular orbital (FMO) analysis was performed. The energy gap between HOMO (highest occupied molecular orbital) and LUMO (lowest unoccupied molecular orbital) was calculated to assess electron transfer capability, with smaller gaps indicating higher reactivity.

#### 3.4.2. Molecular Docking

The 2D structure files of compounds **4**, **7**, and **8** were downloaded from the PubChem database (https://pubchem.ncbi.nlm.nih.gov/) as SDF files. These structures were then converted to 3D and optimized using the MMFF94s force field with the Avogadro v1.2.0 software. The ligand files were subsequently imported into the AutoDockTools v1.5.6 software to be saved as PDBQT files. For protein preparation, the 3D structures of COX-2 and PDE4B proteins were downloaded from the RCSB PDB (https://www.rcsb.org/) with codes 5KIR and 3W5E, respectively [24,25]. Ions, crystallographic waters, and co-crystallized molecules were removed from these protein structures. Missing protein atoms were added, and the structures were saved as PDBQT files using the AutoDockTools v1.5.6 software [25,26].

Validation of docking protocol: To ensure the reliability of the docking procedure, the co-crystallized ligands present in the original PDB structures were used for validation. For COX-2 (5KIR), the native ligand rofecoxib was re-docked into its binding site. Similarly, for PDE4B (3W5E), the native ligand NVW (N-[[3-[(3,4-dimethoxyphenyl)methyl]phenyl]methyl]-3,4-dimethoxybenzenemethanamine) was re-docked. The docking procedure was performed using the same parameters as for the test compounds. The Root mean square deviation (RMSD) between the docked conformation and the original crystallographic pose was calculated [26,27,28]. The obtained RMSD values were 1.32 Å for rofecoxib in COX-2 (5KIR) and 1.58 Å for NVW in PDE4B (3W5E), both below the 2.0 Å threshold, confirming that the docking protocol could accurately reproduce the experimentally observed binding mode. AutoDock Vina v1.2.3 program was used for all docking calculations. The exhaustiveness parameter was set to 400 to ensure high accuracy in docking predictions. All other parameters were kept at their default values. The grid box size was set to 24 Å × 24 Å × 24 Å with a spacing value of 1 Å. The grid box was positioned in the active site of COX-2 at X = 23.3, Y = 0.4, Z = 34.4 (Å), and for PDE4B at X = 24.4, Y = 18.3, Z = −18.5 (Å). Discovery Studio Visualizer software was used to analyze the docking results of compounds **4**, **7**, and **8** with the COX-2 and PDE4B proteins [27,28,29,30].

#### 3.4.3. Molecular Dynamics

All MD simulations were performed using the GROMACS v2023 program. The AMBER99SB-ILDN force field was used to describe the characteristics of the COX-2 and PDE4B proteins. To parameterize the compounds under study, the GAFF2 force field was applied by the Ambertools23 program. Accordingly, the compounds were energy minimized at the B3LYP/6-31G** theoretical level to calculate atomic charges using Gaussian 09 software. The RESP method was used to assign atomic charges to the compounds. TIP3P water molecules in a triclinic box were used to solvate the complexes. To neutralize the complexes, Na^+^ and Cl^−^ counterions were added to achieve electrostatic balance. The solvated complexes were energy minimized using the steepest descent algorithm. The complexes were equilibrated to a temperature of 300 K over 100 ps through the NVT ensemble. Next, the complexes were equilibrated for over 2 ns in the NPT ensemble with the Berendsen barostat applied to maintain pressure. The production phase involved 100 ns MD simulations for all systems. RMSD analyses of protein and ligand in the complexes were conducted using XMGRACE software [27,28]. The 100 ns simulation time was selected based on common practice in the literature for initial stability assessment of protein–ligand complexes. This duration was sufficient for all systems to reach a stable equilibrium, as evidenced by the plateau in the RMSD of the protein backbone typically occurring within the first 20–40 ns. The primary aim of these simulations was to evaluate the overall stability and binding persistence of the complexes. While more advanced analyses—such as hydrogen bond occupancy, per-residue root mean square fluctuation (RMSF) at the binding site, and binding free energy estimation using methods like MM-GBSA or MM-PBSA—provide deeper mechanistic insights, they were beyond the scope of this initial screening study and are reserved for future targeted investigations.

### 3.5. Antioxidant Assay

#### 3.5.1. In Vitro DPPH Radical Scavenging Assay

The antioxidant activity of the Samples was rapidly assessed using the DPPH free radical scavenging assay. A stock DPPH solution was prepared in 100% DMSO and then diluted in 96% ethanol. Samples were dissolved in 100% DMSO at stock concentrations of 1 mg/mL. On a 96-well microplate, sample solutions were serially diluted and mixed with the DPPH solution to achieve final test concentrations ranging from 200 to 12.5 µg/mL for crude extracts and 50 to 3.1 µg/mL for purified samples. Ascorbic acid (5 mM in 10% DMSO) was used as a standard positive control. After incubation at 37 °C for 30 min in the dark, the optical density (OD) was measured at 515 nm using a Tecan Infinite F50 microplate reader [61].

#### 3.5.2. Lipid Peroxidation Inhibition Assay (TBARS Method)

This test evaluates the capacity of samples to inhibit lipid peroxidation by measuring the reduction in malondialdehyde (MDA), a key end-product formed during the peroxidative degradation of cell membrane lipids. MDA reacts with thiobarbituric acid (TBA) under high temperature to generate a pink-colored trimethine chromophore, which absorbs maximally at 532 nm. The experimental procedure was adapted: 0.5 mL of a mouse brain homogenate (prepared in 5 mM phosphate buffer at a 1:10 *w*/*v* ratio) was mixed with 0.1 mL of the samples and 1.4 mL of phosphate buffer. The mixture was incubated at 310 K for 15 min. The reaction was stopped by adding 1 mL of 10% trichloroacetic acid (TCA), followed by centrifugation at 10,000 rpm. A 2.0 mL portion of the clear supernatant was then heated with 1.0 mL of 0.8% TBA in a boiling water bath (373 K) for 15 min. After cooling, the absorbance was read at 532 nm. A buffer blank was used for baseline correction. Trolox (a water-soluble vitamin E analog) was employed as the positive control [62].

#### 3.5.3. Data Analysis for Antioxidant Assays

The percentage of antioxidant activity (AA%) for both the DPPH and TBARS assays was determined using the following formula (4):

AA% = [(Abs_control_ − Abs_sample_)/Abs_control_] × 100 (4)

where Abs_control_ represents the absorbance of the control reaction, which contains all reagents, except for the antioxidant (test sample or positive control). Abs_sample_ represents the absorbance of the reaction mixture, containing the antioxidant, which could be either the test compound (crude extract or purified compound) or a standard positive control (e.g., ascorbic acid).

The antioxidant potency was further characterized by calculating the half-maximal inhibitory concentration (IC_50_). The IC_50_ value denotes the concentration of the antioxidant required to scavenge 50% of the DPPH radicals or to inhibit 50% of lipid peroxidation. This value was derived from a dose–response curve plotting the sample concentration against the corresponding percentage of antioxidant activity. All experiments were performed in at least triplicate, and results are presented as mean ± standard deviation [61,62].

### 3.6. Cell Culture

The murine macrophage cell line RAW 264.7 was obtained from the American Type Culture Collection (ATCC, Manassas, VA, USA). Cells were routinely cultured in Dulbecco’s Modified Eagle Medium (DMEM) supplemented with 10% (*v*/*v*) heat-inactivated fetal bovine serum (FBS), 100 U/mL penicillin, and 100 µg/mL streptomycin. All cell culture media and supplements were purchased from Thermo Fisher Scientific (Waltham, MA, USA), unless otherwise specified. Cells were maintained in a humidified incubator at 310 K with a 5% CO_2_ atmosphere and subcultured every 2–3 days upon reaching 80–90% confluency using a cell scraper. For experiments, cells were seeded in appropriate multi-well plates at a desired density and allowed to adhere overnight in complete growth medium prior to treatment. Reagents for Bioassays: Lipopolysaccharide (LPS), MTT (3-(4,5-dimethylthiazol-2-yl)-2,5-diphenyltetrazolium bromide), and Griess reagent were purchased from Merck KGaA (Darmstadt, Germany) [63,64].

### 3.7. Evaluation of Anti-Inflammatory Activity via NO Production Assay

The inhibitory effect on NO generation was investigated in an LPS-induced inflammation model using RAW 264.7 macrophages. After pre-treatment with experimental samples, cells were stimulated with LPS (1 µg/mL) for 24 h. NO levels in supernatants were quantified as stable nitrite derivatives using Griess reagent, with spectrophotometric detection at 540 nm. Cardamonin served as the reference inhibitor. Cell viability was assessed using the MTT assay at all tested concentrations to ensure that the inhibition of NO and cytokine production was not due to cytotoxicity [63,64].

### 3.8. Assessment of Pro-Inflammatory Cytokine Secretion

The ability of test samples to inhibit the release of major pro-inflammatory cytokines was determined by ELISA. Following the experimental setup described for the NO assay (Section 3.6), RAW 264.7 cells were co-treated with samples and LPS (1 µg/mL) for 24 h. Cell culture supernatants were then harvested, clarified by centrifugation, and analyzed for secreted mouse TNF-*α* and IL-6 using commercial ELISA kits (Abcam) in strict accordance with the provided protocols. Absorbance was measured at 450 nm, and cytokine concentrations were derived from standard curves. The percentage inhibition of cytokine secretion was calculated in comparison to LPS-stimulated control cells [65,66].

### 3.9. Statistical Analysis

Data from all in vitro experiments are expressed as the mean ± standard deviation (SD) of at least three independent replicates. Statistical comparisons between experimental groups were performed using one-way analysis of variance (ANOVA), followed by Tukey’s post hoc test for multiple comparisons in GraphPad Prism software (version 9.5.1).

## 4. Conclusions

The study successfully isolated and identified eight compounds from *P. zeylanica*, including bioactive triterpenoids and flavonoids. The main findings are summarized as follows:

Compound **3** showed superior in vitro antioxidant activity, with the lowest IC_50_ values in both DPPH (18.52 ± 1.50 µM) and TBARS (10.34 ± 0.93 µM) assays. In silico DFT calculations supported this finding, revealing favorable thermodynamic parameters (low BDE and PA values) and elucidating the PT mechanism in solvents as a key antioxidant pathway for this compound.

Compound **8** exhibited the most potent in vitro anti-inflammatory effect, demonstrating strong inhibition of NO production (IC_50_: 16.25 ± 0.95 µM) as well as the secretion of pro-inflammatory cytokines TNF-*α* (IC_50_: 12.97 ± 0.88 µM) and IL-6 (IC_50_: 22.52 ± 1.98 µM).

Compounds **4**, **7**, and **8** demonstrated favorable binding affinity to COX-2 and PDE4B enzymes in molecular docking studies, suggesting their anti-inflammatory action may involve the inhibition of these key enzymatic pathways. Molecular dynamics simulations further confirmed the stability of these protein–ligand complexes over 100 ns.

These comprehensive results, integrating in vitro bioassays with in silico computational analyses (DFT, docking, and MD simulations), not only validate the medicinal value of *P. zeylanica* but also provide a solid scientific foundation for further research. It should be noted that our in vitro anti-inflammatory findings are based on a murine macrophage line (RAW 264.7). Further validation using human primary cells or in vivo inflammation models is necessary to fully generalize the anti-inflammatory potential of these lead compounds and translate the findings toward therapeutic applications. This includes structural optimization of the lead compounds, in vivo pharmacological evaluation, and the development of functional foods or therapeutic products derived from these promising natural compounds.

## Figures and Tables

**Table 1:** The enthalpies at 298K for radicals of **1** and **3** at B3LYP/6-31+G(d,p) level of theory (in kcal/mol).

No	BDE			IP			PA		
	Gas	Water	DMSO	Gas	Water	DMSO	Gas	Water	DMSO
**1**-C12-H	105.0	104.1	104.4	170.2	111.6	116.9	362.5	106.1	86.3
**1**-3-OH	98.0	97.8	97.8				366.3	70.0	49.8
**3**-C12-H	104.7	104.0	103.9	169.1	112.5	118.1	401.5	108.4	88.5
**3**-2-OH	96.5	97.1	96.9				363.6	68.2	48.3
**3**-3-OH	96.5	96.6	96.3				360.0	67.4	47.2

**Table 2:** Binding affinity (in kcal/mol) and amino acid interactions for the compounds **4**, **7**, and **8** against the COX-2 and PDE4B proteins compared to reference ligands.

Compounds	PDB ID	Binding Affinity (kcal/mol)	Hydrogen Bond	Hydrophobic Interaction
4	5KIR	−6.265	-	His351
	3W5E	−6.146	Gln284, Asn283, Tyr233, Asp392	His278, His234, Phe414, Phe446
7	5KIR	−6.957	Tyr355, Gln192	His351, Lys358
	3W5E	−9.63	His234, Val281, Glu509	Phe446, Tyr233, Phe506
8	5KIR	−7.17	Tyr355, Gly354, Asn350, His356, Asp347	Lys358, His351
	3W5E	−9.74	Glu304, Tyr233	Phe446, Ser282
rofecoxib	5KIR	−8.9	Arg513	Leu352, Val349, Val523, Ser353, Ala527, His90
NVW	3W5E	−12.05	Gln443	Ile410, Met503, Leu502, Phe414, Met347

## Data Availability

The original contributions presented in this study are included in the article. Further inquiries can be directed to the corresponding authors.

## Supplementary Materials

The following supporting information can be downloaded at: https://www.mdpi.com/article/10.3390/molecules31020357/s1, Figure S1: The NMR data of compounds **1**-**8**; Figure S2: Dose-response curves of the most active key compounds in bioactivity assays.
