# Supplementary material for: In Silico and In Vitro Insights into the Pharmacological Potential of Pouzolzia zeylanica
Source: Molecules. 2026 Jan 20;31(2):357. doi: 10.3390/molecules31020357 (PMC12843715; doi:10.3390/molecules31020357)
Supplement: Supplementary file 1 [file molecules-31-00357-s001.zip › molecules-4065208-supplementary.pdf]

Article

In silico and *in vitro* insights into the pharmacological potential of *Pouzolzia zeylanica*

Nguyen Anh Hung[1], Vu Thi Thu Le[2], Nguyen Viet Hung[3], Ha Thi Minh Tam[4], Nguyen Ngoc Linh[5], Nguyen Quang Hop[1], Nguyen Thi Hanh[6], Do Tien Lam[7,8*]

[1] Faculty of Chemistry, Hanoi Pedagogical University 2, Phuc Yen, Phu Tho 35000, Vietnam
[2] Thai Nguyen University of Agriculture and Forestry (TNU), Quyet Thang, Thai Nguyen 250000, Vietnam
[3] Ethnic Institute, Urban Dream Town, Tay Mo, Hanoi 100000, Vietnam
[4] Preschool Education, Hanoi Pedagogical University 2, Phuc Yen, Phu Tho 35000, Vietnam
[5] Faculty of Training Bachelor of Practice, Thanh Do University, Kim Chung, Hanoi 100000, Vietnam
[6] Center for high technology research and development (VAST), 18 Hoang Quoc Viet, Nghia Do, Hanoi 100000, Vietnam
[7] Institute of Chemistry (VAST), 18 Hoang Quoc Viet, Nghia Do, Hanoi 100000, Vietnam
[8] Faculty of Chemistry, Graduate University of Science and Technology (VAST), 18 Hoang Quoc Viet, Nghia Do, Hanoi 100000, Vietnam
Correspondence: dotienlam198@gmail.com,(+84)989856515

Figure S1. The NMR data of compounds 1-8

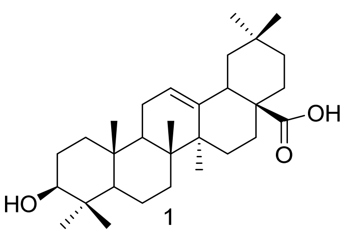

1

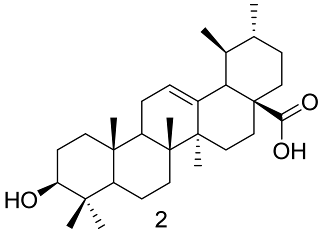

2

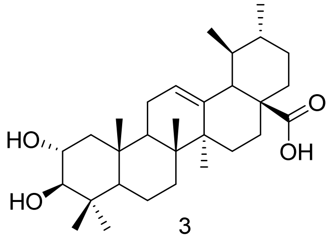

3

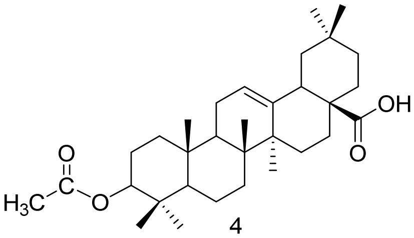

4

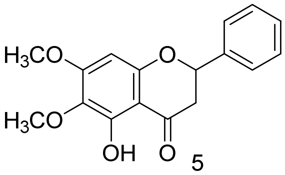

5

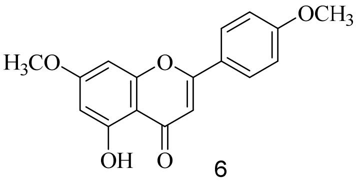

6

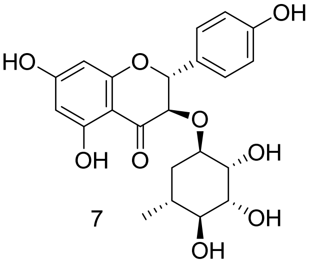

7

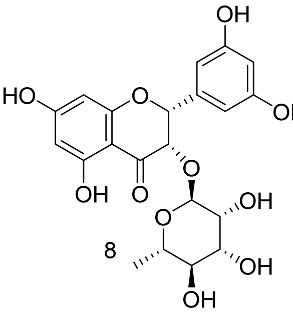

8

• ***Oleanolic acid (1)***

^{1}H-NMR (500 MHz, $CDCl_3$), δ(ppm): 5.27 (1H, H-12); 3.22 (1H, dd, *J* =11,0 và 4,0Hz, H-3); 2.82 (1H, dd, *J*= 8.5 và 4.0Hz, H-18); 1.13 (3H, s, 27-CH_3), 0,98 (3H, s, 25-CH_3); 0,92 (3H, s, 30-CH_3); 0,91 (3H, s, 29-CH_3); 0.90 (3H, s, 23-CH_3); 0,77 (3H, s, 24-CH_3); 0,75 (3H, s, 26-CH_3).

^{13}C-NMR (125 MHz, $CDCl_3$), δ(ppm): 183,2 (C-28); 143,6 (C-13); 122,6 (C-12); 79,1 (C-3); 55,2 (C- 5); 47,7 (C -9); 46.5 (C-17); 45.9 (C-19); 41,6 (C-14); 41.0 (C-18); 39.3 (C-8); 38.8 (C-4); 38.4 (C-1); 37.1 (C-10); 33.8 (C-21); 33.1 (29-CH_3); 32.6 (C-7); 32.5 (C-22); 30.7 (C-20); 28.1 (23-CH_3); 27.7 (C-15); 27.2 (C-2); 25.9 (27-CH_3); 23.4 (30-CH_3); 22.8 (C-16); 22.9 (C-11); 18.3 (C-6); 17.1 (26-CH_3); 15.6 (24-CH_3); 15.3 (25-CH_3).

• ***Ursolic acid (2)***

^{1}H-NMR (500 MHz, MeOD), δ(ppm): 5.23 (1H, t, *J* = 3.5 and 7.5 Hz, H-12), 3.17 (1H, dd, *J* = 5.0 and 11.5 Hz, H-3), 2.20 (1H, d, *J* = 11.5 Hz, H-18), 1.94 (1H, m, H-6), 1.12 (3H, s, H-27), 0.98 (3H, s, H-23), 0.97 (3H, d, *J* = 6.5 Hz, H-29), 0.95 (1H, s, H-25), 0.89 (3H, d, *J* = 6.5 Hz, H-30), 0.85 (3H, s, H-26), 0.78 (3H, s, H-24), 0.76 (1H, d, *J* = 11.5 Hz, H-5).

^{13}C-NMR (125 MHz, MeOD), δ(ppm): 40.0 (C-1), 27.91 (C-2), 79.7 (C-3), 39.8 (C-4). 56.7 (C-5), 19.5 (C-6), 34.3 (C-7), 40.8 (C-8), 49.2 (C-9), 38.1 (C-10), 24.4 (C-11), 126.9 (C-12), 139.6 (C-13), 43.3 (C-14), 29.2 (C-15), 25.3 (C-16), 49.2 (C-17), 54.4 (C-18), 40.4 (C-19), 40.4 (C-20), 31.8 (C-21), 38.1 (C-22), 28.8 (C-23), 16.4 (C-24), 16.0 (C-25), 17.8 (C-26), 24.1 (C-27), 180.6 (C-28), 21.6 (C-29), 17.6 (C-30).

• ***2α- hydroxy-ursolic acid (3)***

^{1}H-NMR (500 MHz, $CDCl_3$-MeOD), δ(ppm): 5.13 (1H, d, *J* = 14.6 Hz, H-12), 3.40 (1H, dd, *J* = 4.2 và 13.7 Hz, H-2), 2.73 (1H, d, *J* = 9.3 Hz, H-3), 2.10 (1H, d, *J* = 11.2 Hz, H-18), 1.03 (3H, s, H-27), 0.91 (3H, s, H-23), 0.90 (3H, s, H-25), 0.89 (3H, d, *J* = 4.0 Hz, H-29), 0.80 (3H, d, *J* = 6.4 Hz, H-30), 0.73 (3H, s, H-24), 0.69 (3H, s, H-26).

^{13}C-NMR (125MHz, $CDCl_3$-MeOD), δ (ppm): 47.0 (C-1), 67.1 (C-2), 82.2 (C-3), 45.3 (C-4), 52.3 (C-5), 18.0 (C-6), 32.6 (C-7), 38.8 (C-8), 46.9 (C-9), 37.5 (C-10), 22.7 (C-11), 124.4 (C-12), 138.2 (C-13), 41.6 (C-14), 27.4 (C-15), 23.7 (C-6), 46.8 (C-17), 54.7 (C-18), 38.4 (C-19), 38.4 (C-20), 30.1 (C-21), 36.2 (C-22), 28.8 (C-23), 16.9 (C-24), 16.4 (C-25), 16.9 (C-26), 23.3 (C-27), 178.2 (C-28), 21.0 (C-29), 17.1 (C-30).

• ***3β-O-acetyl-12-oleanen-28-oic acid (4)***

^{1}H-NMR (500 MHz, $CDCl_3$), δ (ppm): 5.27 (1H, H-12), 4.49 (1H, td, J 15.9; 9.4; vμ 6.5Hz, H-3), 2.82 (1H, dd, J 13.7 vμ 3.7Hz, H-18), 2.03 (3H, s, O**CH**$_3$), 1.98 (1H, ddd, 13.7 vμ 3.9Hz, H-16), 1.31 (3H, s, 27-CH_3), 0.94 (3H, s, 25-CH_3), 0.93 (3H, s, 30-CH_3), 0.90 (3H, s, 29-CH_3), 0.86 (3H, s, 23-CH_3), 0.85 (3H, d, J 9.3Hz, 24-CH_3), 0.75 (3H, s, 26-CH_3).

^{13}C-NMR (125 MHz, $CDCl_3$), δ (ppm): 183.2 (C-28), 170.9 ($OCOCH_3$), 143.7 (C-13), 122.6 (C-12), 81.0 (C-3), 55.4 (C- 5), 47.7 (C -9), 46.6 (C-17), 45.9 (C-19), 41.7 (C-14), 41.1 (C-18), 39.4 (C-8), 38.2 (C-1), 37.8 (C-4), 37.1 (C- 10), 33.9 (C-21), 33.1 (29-CH_3), 32.7 (C-7), 32.5 (C-22), 30.7 (C-20), 28.1 (23-CH_3), 27.8 (C-15), 25.9 (27-CH_3), 23.6 (C-2), 23.5 (C-16), 23.0 (C-11), 21.3 ($OCOCH_3$), 18.3 (C-6), 17.2 (26-CH_3), 16.7 (24-CH_3), 15.4 (25-CH_3)

• ***5-hydroxy-6,7-dimetoxyflavanon (5)***

^{1}H-NMR (500MHz, $CDCl_3$), δ (ppm): 5.47 (1H, dd, 11.0 and 2.5 Hz, H-2); 3.04 and 2.80 (2H, dd, 13.5 và 2.5 Hz, H-3); 6.21 (1H, s, H-8); 7.48 (2H, m, H-2' và H-6'); 7.43 (2H, m, H-3' và H-5'); 7.37 (1H, m, H-4'); 3.87 (3H, s, 6-OCH_3); 3.88 (3H, s, 7-OCH_3).

Phổ ^{13}C-NMR (125MHz, $CDCl_3$), δ(ppm): 79.6 (C-2); 45.7 (C-3); 189.0 (C-4); 155.5 (C-5); 128.6 (C-6); 158.2 (C-7); 92.3 (C-8); 155.4 (C-9); 106.1 (C-10); 138.7 (C-1'); 125.9 (C-2' and C-6'); 128.8 (C-3' and C-5'); 128.5 (C-4'); 61.5 (6-OCH_3); 56.2 (7-OCH_3).

• ***4′-methoxytectochrysin (6)***

^{1}H-NMR (500 MHz, CD^3OD); (ppm): 12.81 (5-OH), 7.85 (2H, dd, 1.5 và 5.5 Hz, H-2' and H-6'); 7.03 (2H, dd, 1.5 and 5.5 Hz, H-3' and H-5'); 6.58 (1H, s, H-3); 6.49 (1H, d, 2.0 Hz, H-8); 6.37 (1H, d, 2.0 Hz, H-6); 3.89 (3H, s, 4'-OCH_3); 3.88 (3H, s, 7-OCH_3).

^{13}C-NMR (125 MHz, CD_3OD); ◎ (ppm): 164.1 (C-2); 104.4 (C-3); 182.5 (C-4); 162.3 (C-5); 98.1 (C-6); 165.5 (C-7); 92.7 (C-8); 157.8 (C-9); 105.6 (C-10); 123.7 (C-1'); 128.1 (C-2', C-6'); 162.6 (C-4'); 114.5 (C-3', C-5'); 55.8 (3'-OCH_3); 55,6 (7-OCH_3).

• ***3,4′,5,7-tetrahydroxyflavanone-3-O-L-rhamnopyranoside (7)***

^{1}H-NMR (500 MHz, MeOD), δ (ppm) : 7.38 (2H, d, *J*= 8,5Hz, H-2′ và H-6′), 6.87 (2H, d, *J*= 8.5 Hz, H-3′ và H-5′), 5.94 (1H, d, *J*= 2.0 Hz, H-6), 5.92 (1H, d, *J*= 2.0, H-8), 5.16 (1H, d, *J*= 10.8 Hz, H-2), 4.63 (1H, d, *J*= 10.8 Hz, H-3), 4.25 (1H, dd, *J*= 3.6 and 10.8 Hz, H-3″), 4.04 (1H, d, *J*= 1.2 Hz, H-1″), 3.67 (1H, dd, *J*= 3.2 and 9.6Hz, H-4″), 3.53 (1H, dd, *J*= 1.3 and 3.7Hz, H-2″), 1.20 (3H, d, *J*= 6.2 Hz, H-6″).

^{13}C-NMR (125 MHz, DMSO-d_6), δ (ppm) : 196.0 (C-4), 168.6 (C-7), 165.5 (C-5), 164.1 (C-9), 159.4 (C-4″), 130.0 (C-2′), 130.0 (C-6′), 128.6 (C-1′), 116.5 (C-3′), 116.5 (C-5′), 102.5 (C-10), 102.2 (C-1″), 97.4 (C-6), 96.3 (C-8), 83.8 (C-2), 78.7 (C-3), 73.8 (C-5″), 72.2 (C-4″), 71.7 (C-2″), 70.5 (C-3″), 17.8 (C-6″).

• ***3,3′,4′,5,7-pentahydroxyflavanone-3-O-L-rhamnopyranoside (8)***

^{1}H-NMR (500 MHz, MeOD), δ (ppm) : 6.97 (1H, d, *J*= 1.8 Hz, H-2′); 6.86 (1H, dd, *J*= 8.2 and 1.8 Hz, H-6′); 6.82 (1H, d, *J*= 8.1Hz, H5′); 5.94 (1H, d, *J*= 2.2Hz, H-6); 5.92 (1H, d, *J*= 2.0 Hz, H-8); 5.10 (1H, d, *J*= 10.6Hz, H-2); 4.58 (1H, d, *J*= 10.7 Hz, H-3); 4.25 (1H, dd, *J*= 3.6 and 6.3 Hz, H-1′); 4.08 (1H, d, *J*= 1.3Hz, H-1″); 3.67 (1H, dd, *J*= 3.3 and 10.6 Hz, H-3″); 3.56 (1H, dd, *J*= 3.1 and 1.6Hz, H-2″); 3.34 (1H, d, *J*= 11.6 Hz, H-4″); 1.20 (3H, d, *J*= 6.3 Hz, H-6″).

^{13}C-NMR (125 MHz, MeOD), δ (ppm) : 195.9 (C-4), 168.6 (C-7), 165.5 (C-5), 164.1 (C-9), 147.4 (C-4′), 146.5 (C-3′), 129.2 (C-1′), 120.5 (C-6′), 116.3 (C-2′), 115.5 (C-5′), 102.5 (C-10), 102.1 (C-1″), 97.4 (C-6), 96.3 (C-8), 83.9 (C-2), 78.5 (C-3), 73.8 (C-4″), 72.2 (C-3″), 71.8 (C-2″), 70.5 (C-5″), 17.8 (C-6″).

Compound oleanolic acid (1)

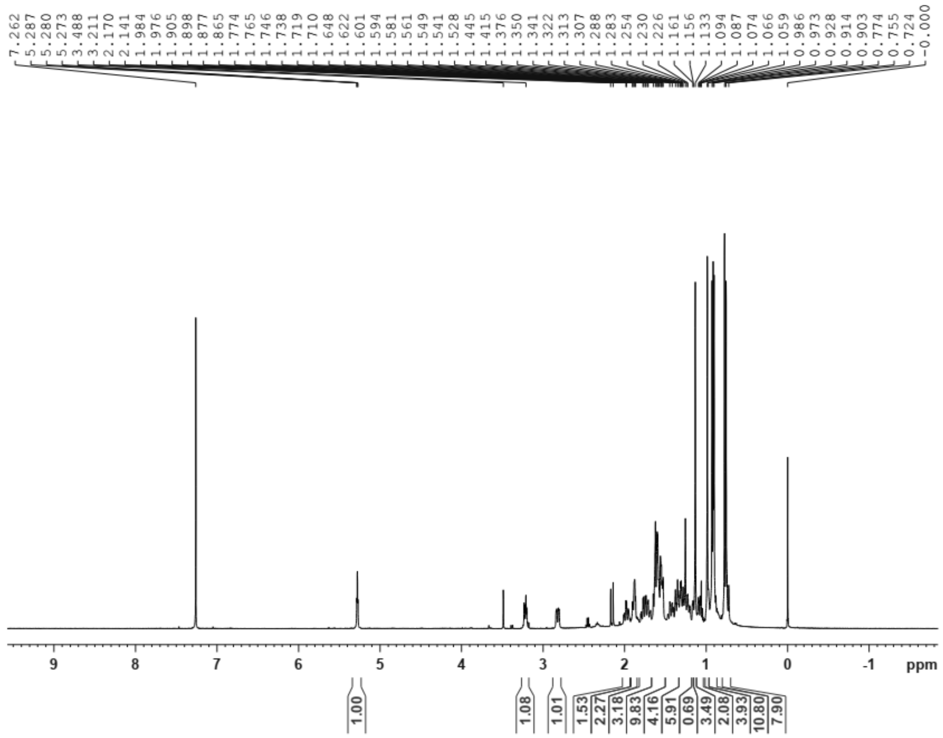

^{1}H-NMR spectrum of **1**

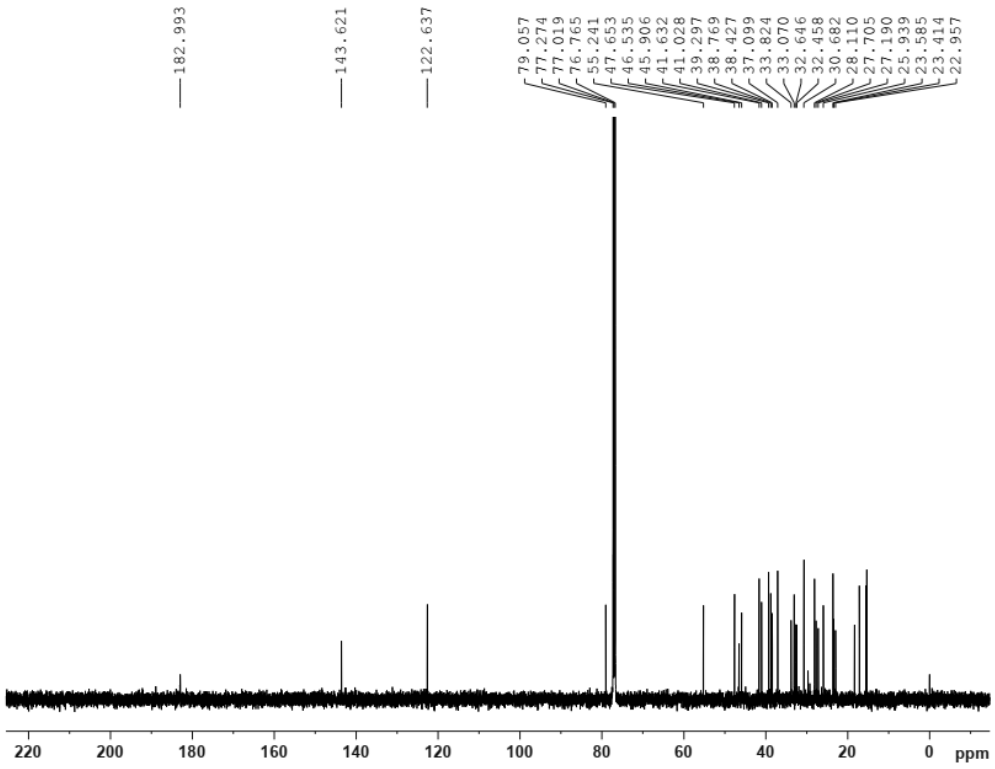

^{13}C-NMR spectrum of **1**

Compound ursolic acid (2)

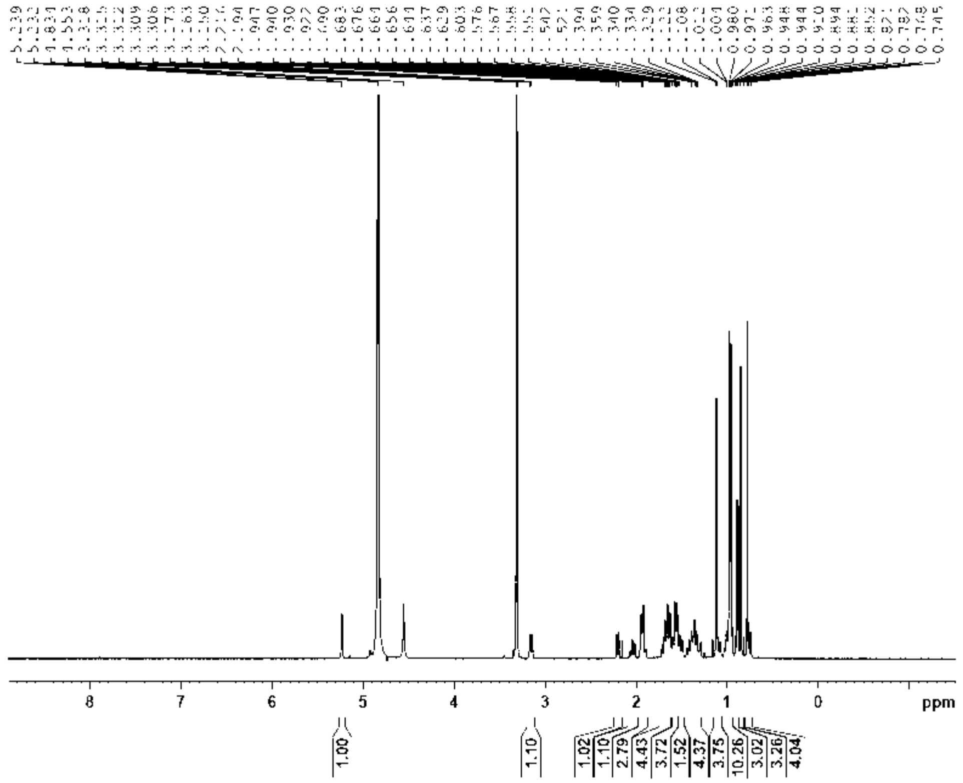

^{1}H-NMR spectrum of **2**

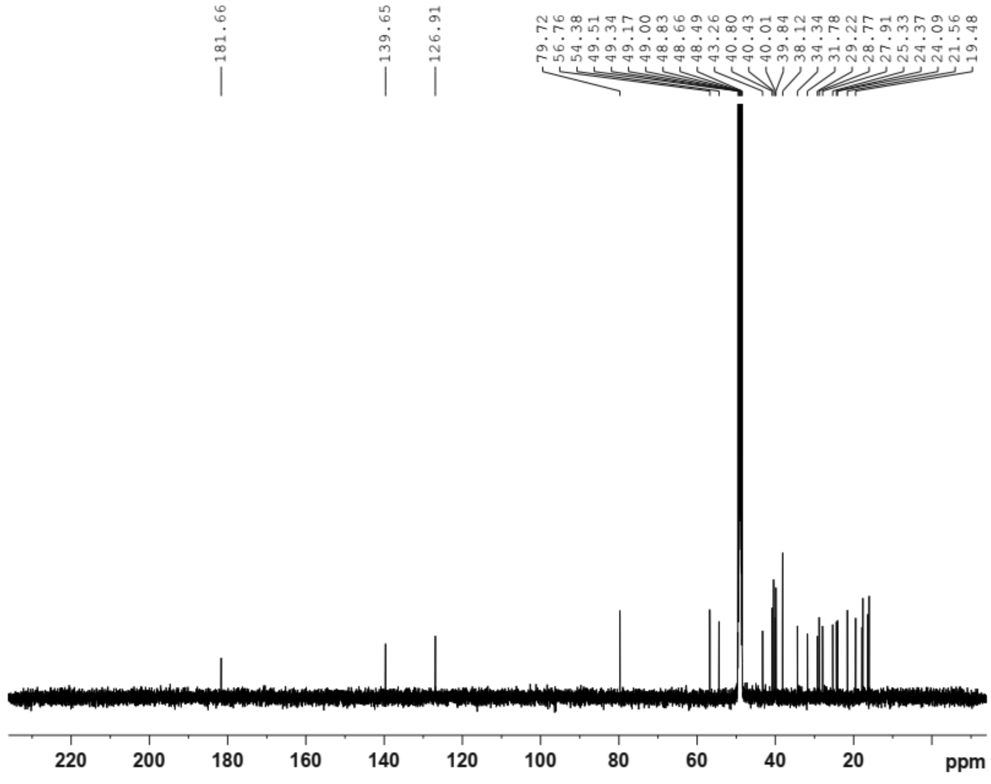

^{13}C-NMR spectrum of **2**

Compound 2α- hydroxy-ursolic acid (3)

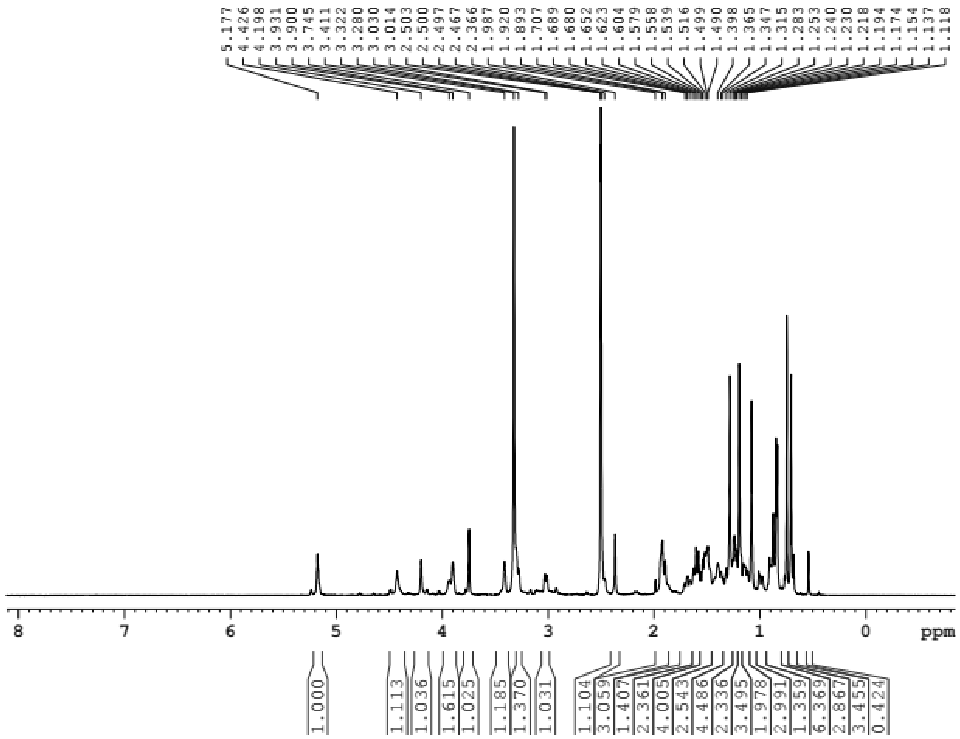

^{1}H-NMR spectrum of **3**

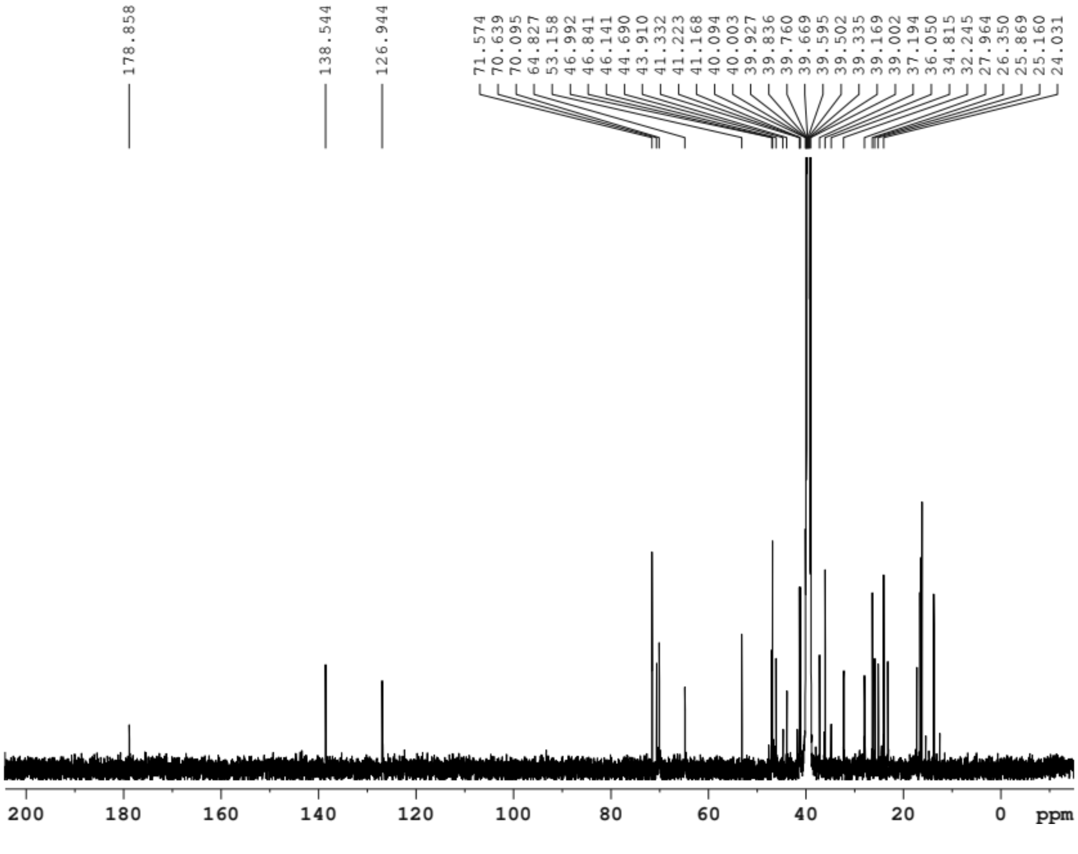

^{13}C-NMR spectrum of **3**

Compound 3β-O-acetyl-12-oleanen-28-oic acid (4)

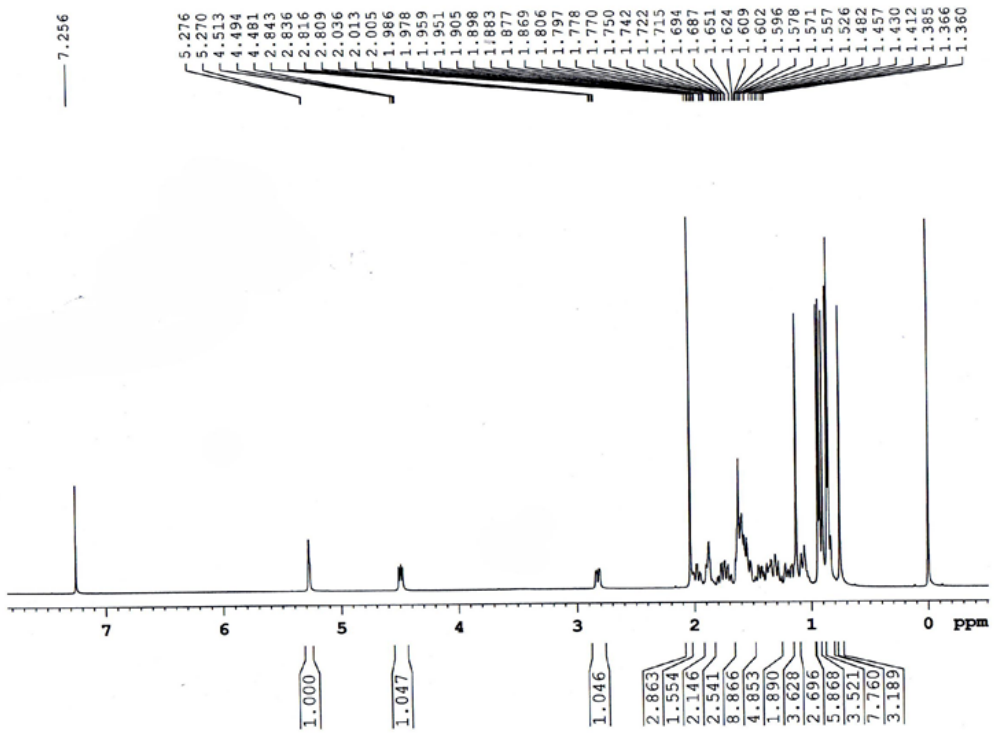

^{1}H-NMR spectrum of **4**

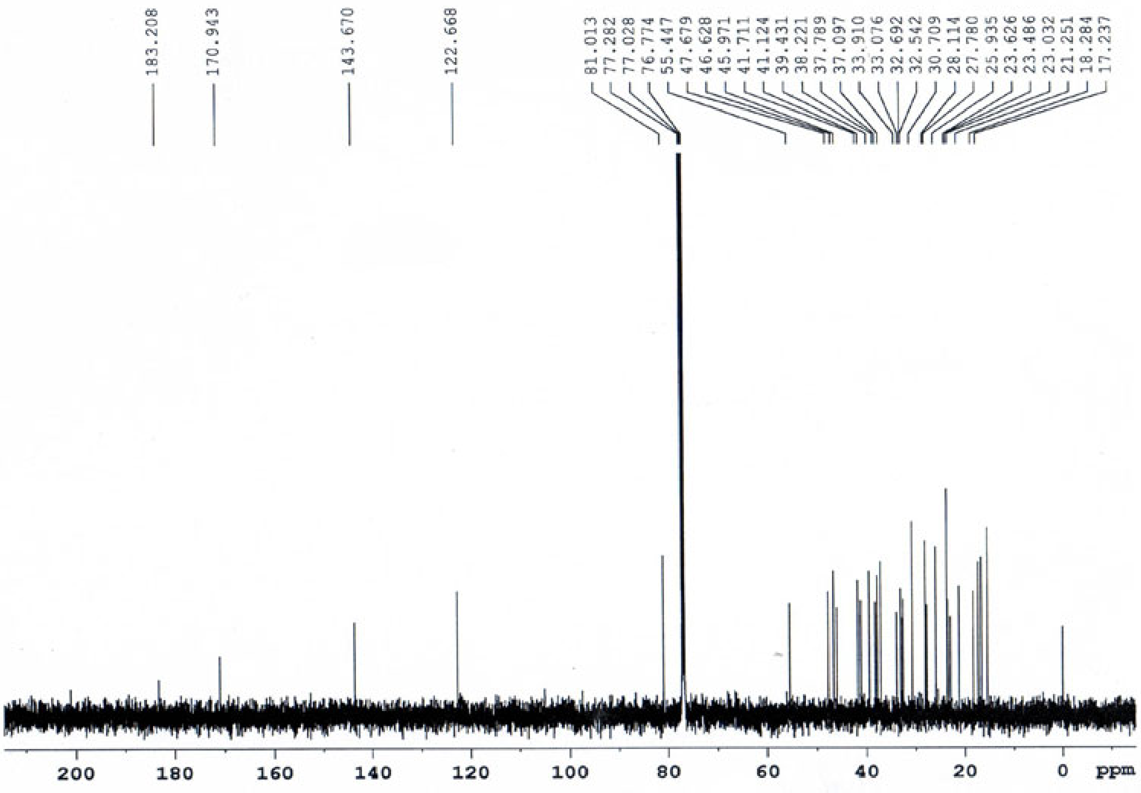

^{13}C-NMR spectrum of **4**

Compound 5-hydroxy-6,7-dimetoxyflavanon (5)

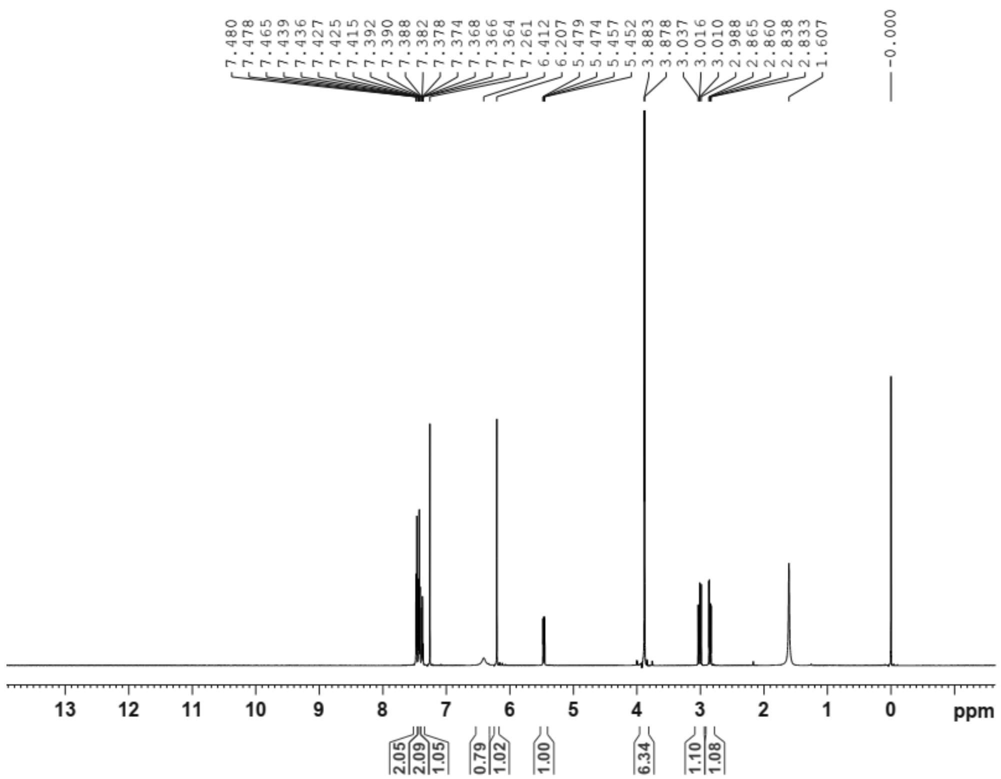

^{1}H-NMR spectrum of **5**

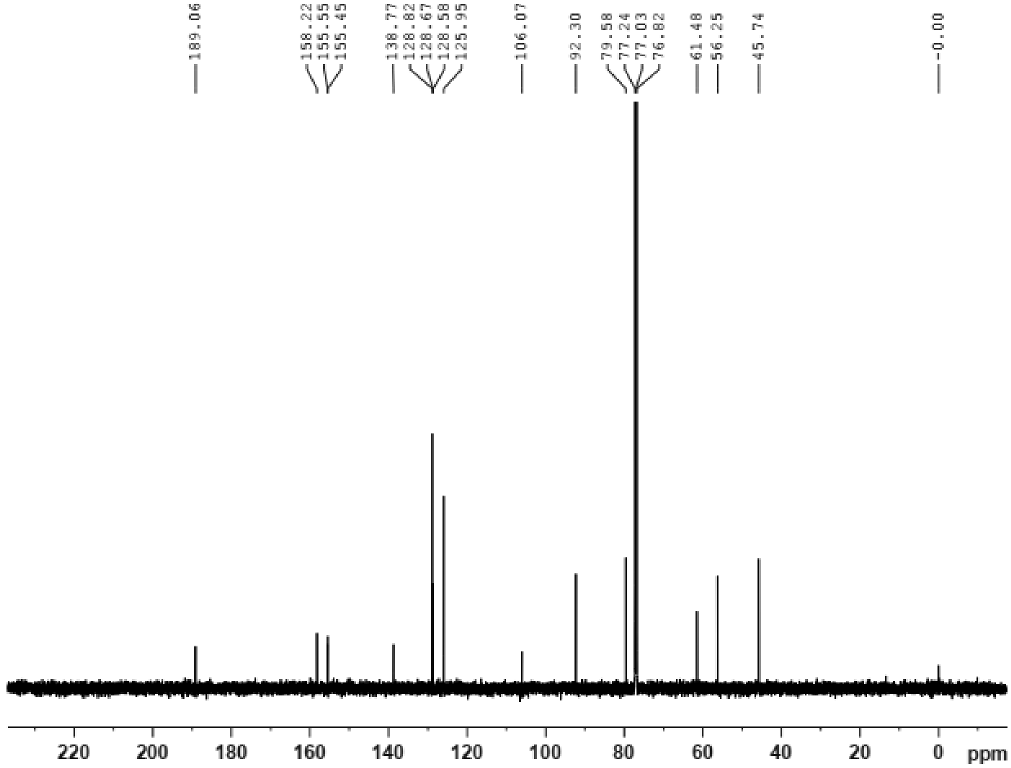

^{13}C-NMR spectrum of **5**

Compound 4′-methoxytectochrysin (6)

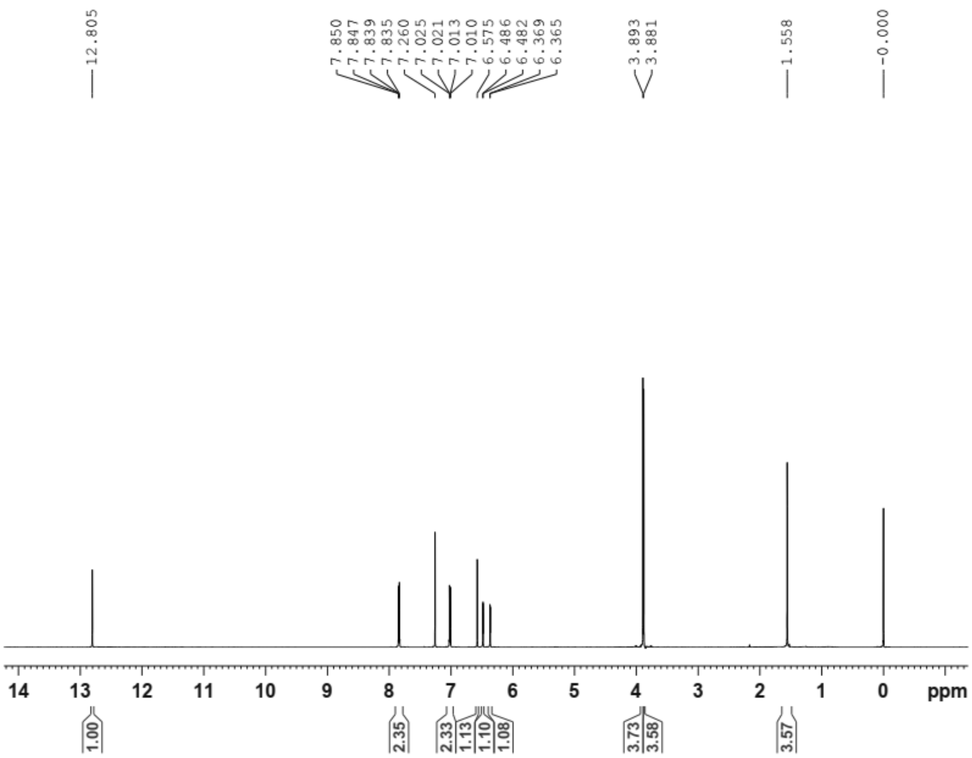

^{1}H-NMR spectrum of **6**

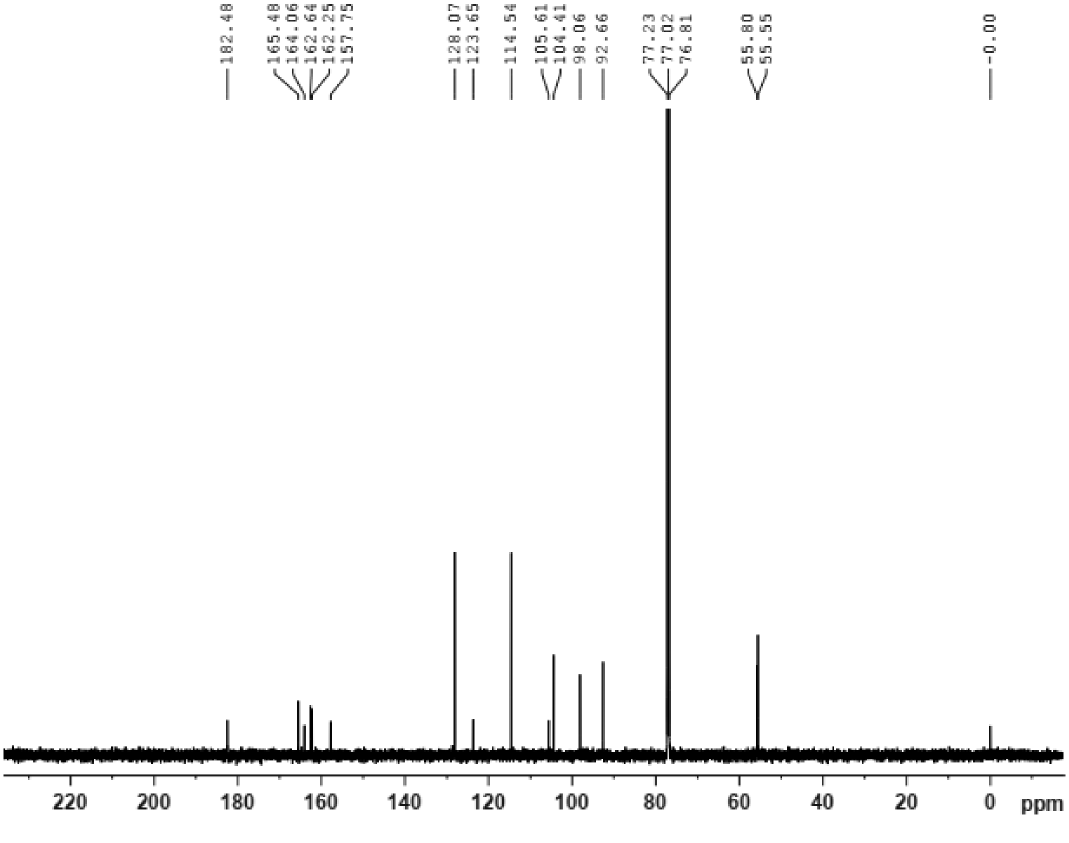

^{13}C-NMR spectrum of **6**

Compound 3,4′,5,7-tetrahydroxyflavanone-3-O-L-rhamnopyranoside (7)

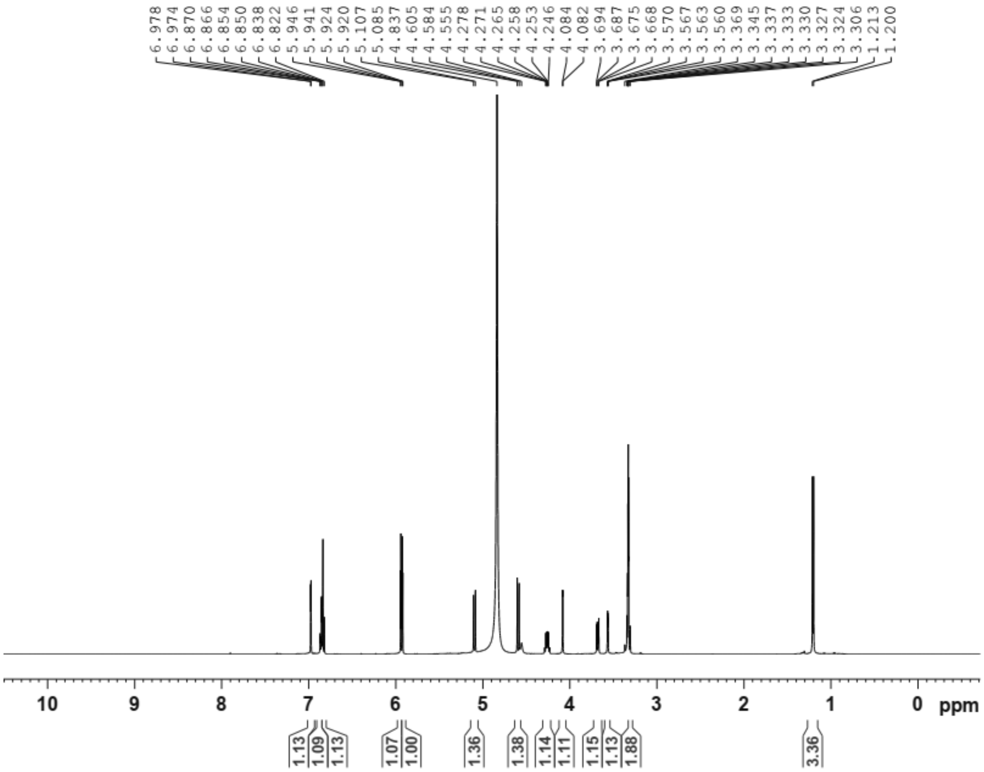

^{1}H-NMR spectrum of **7**

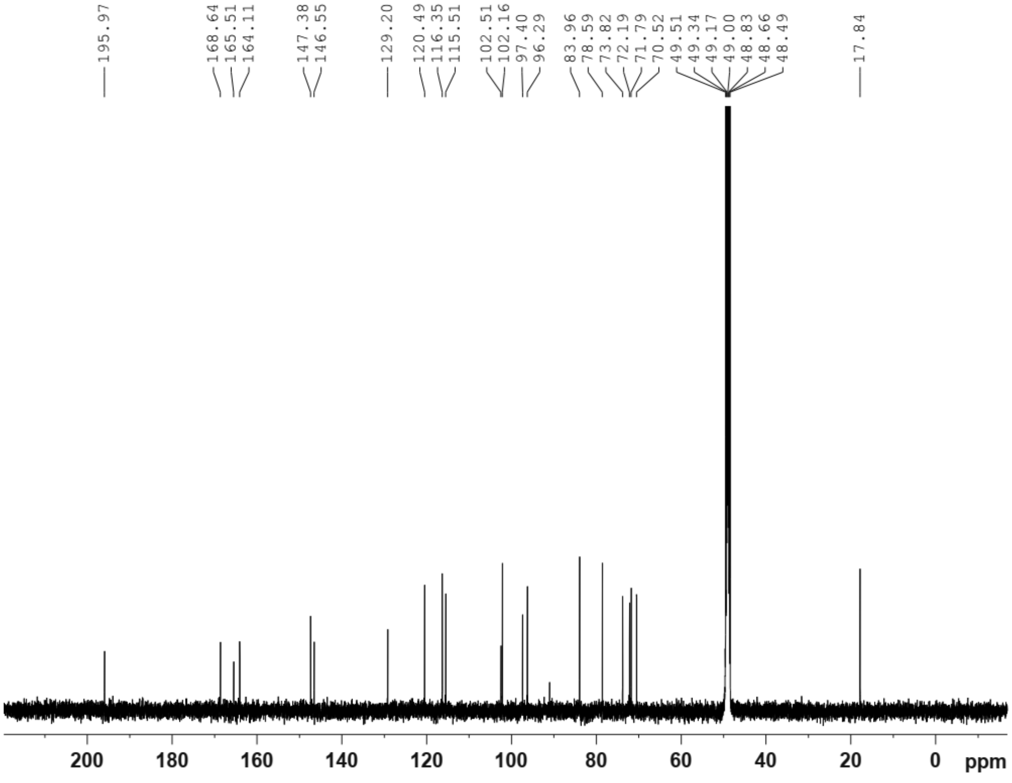

^{13}C-NMR spectrum of **7**

Compound 3,3′,4′,5,7-pentahydroxyflavanone-3-O-L-rhamnopyranoside (8)

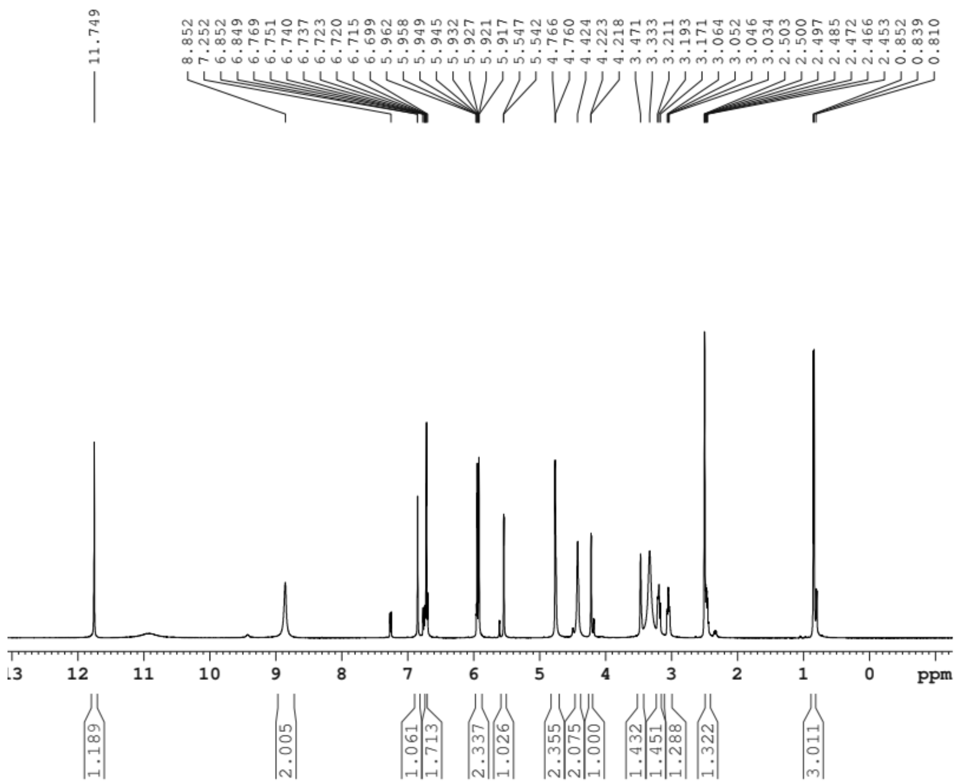

^{1}H-NMR spectrum of **8**

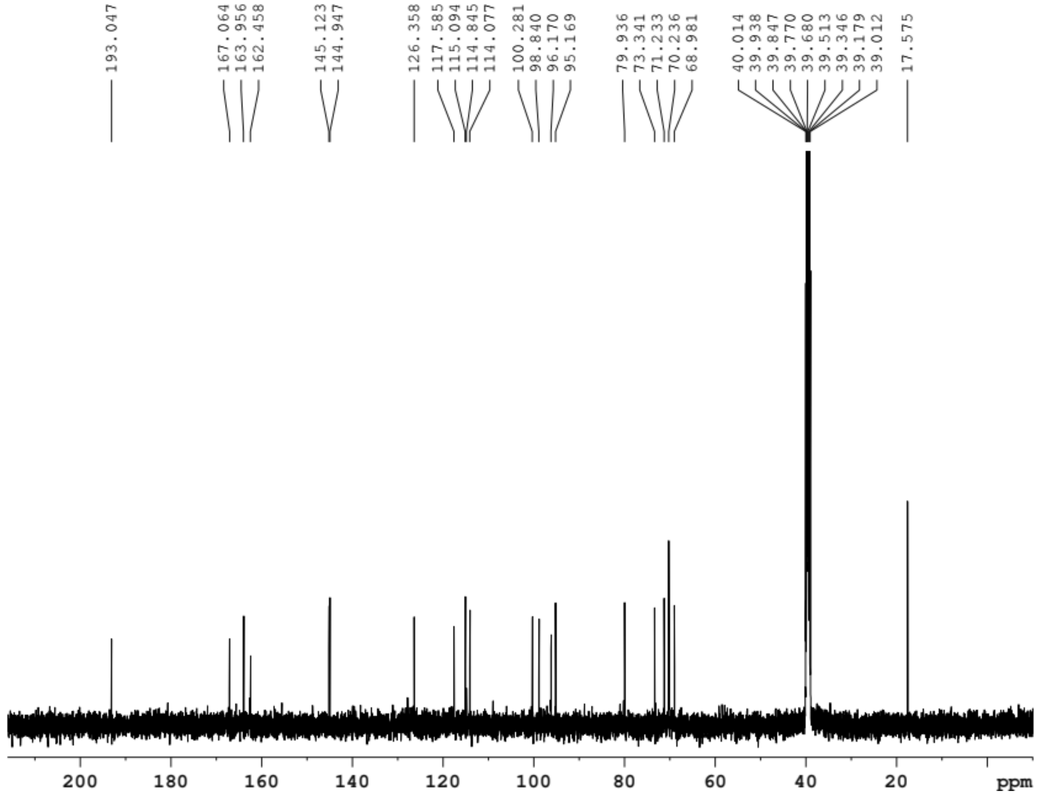

^{13}C-NMR spectrum of **8**

Figure S2. **Dose-response curves of the most active key compounds in bioactivity assays (a, b, c)**

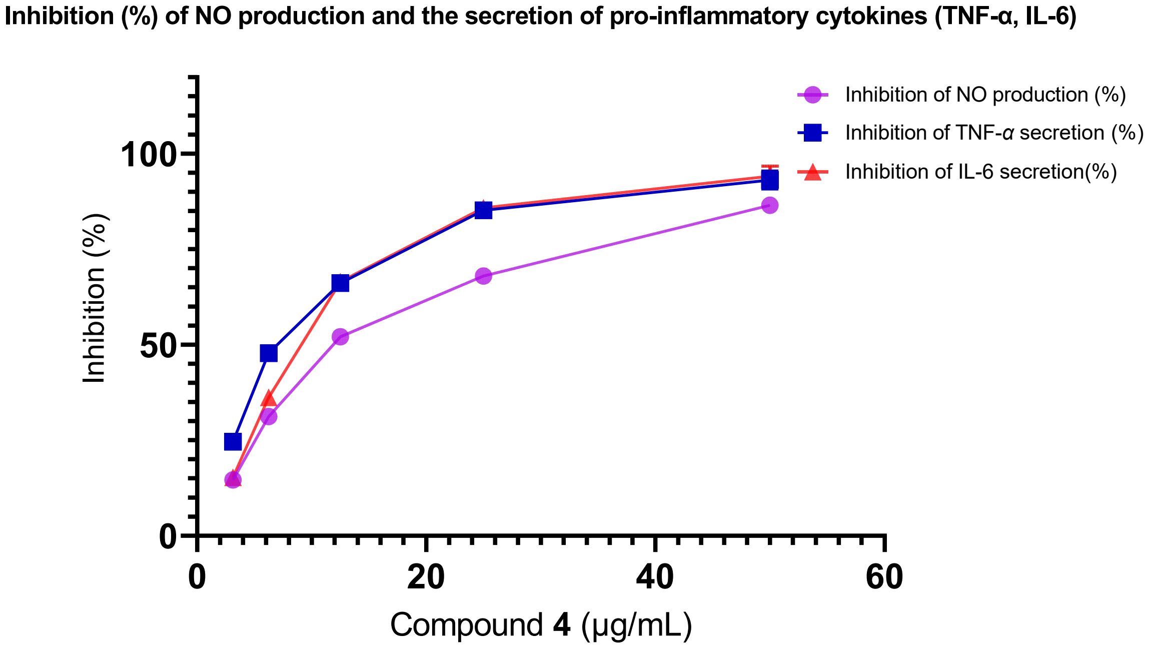

(a)

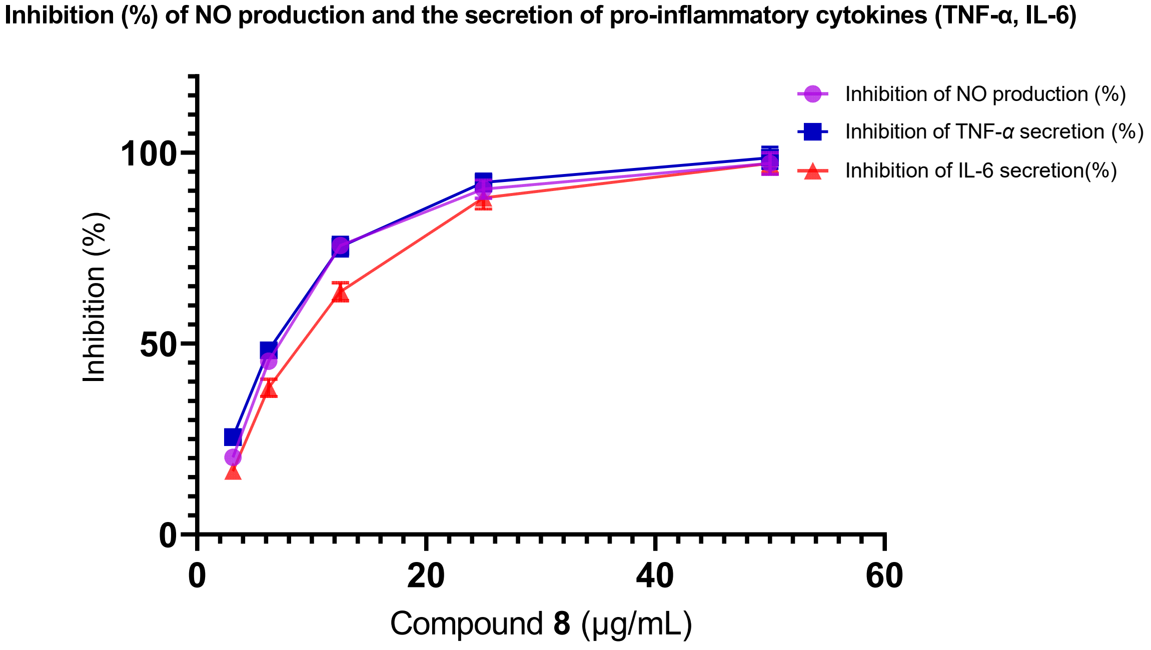

(b)

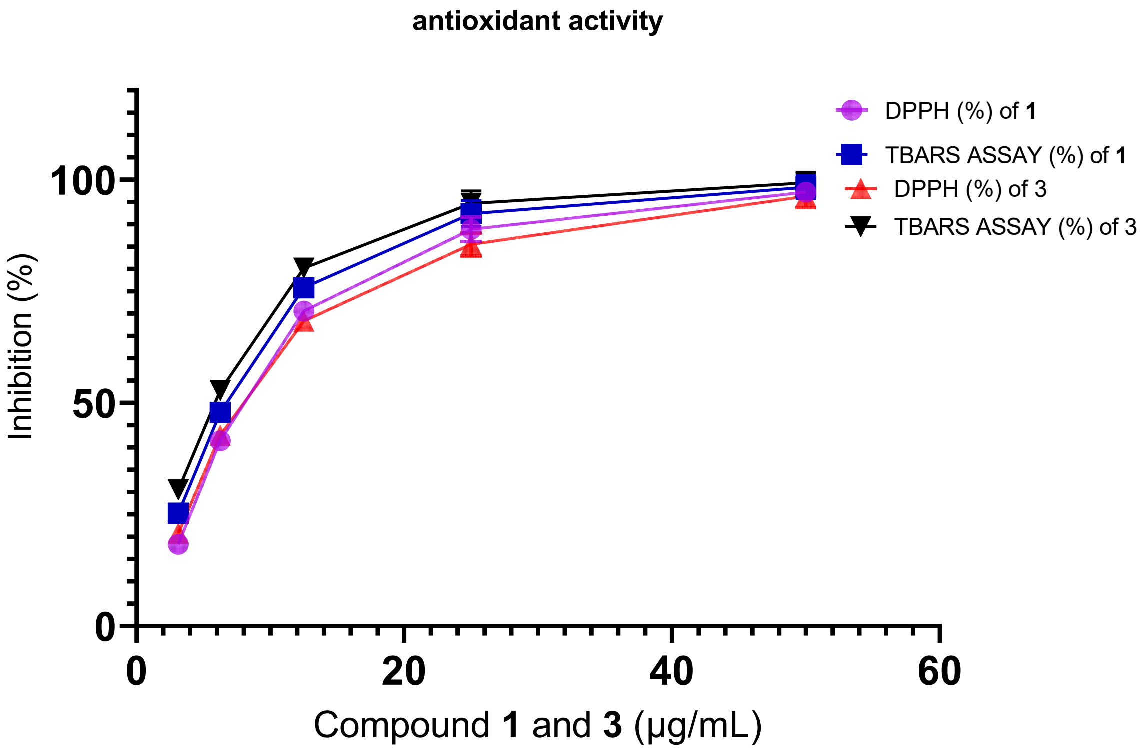
antioxidant activity
DPPH (%) of 1
TBARS ASSAY (%) of 1
DPPH (%) of 3
TBARS ASSAY (%) of 3
Inhibition (%)
100
50
0
0
20
40
60
Compound 1 and 3 (µg/mL)

(c)
